# Supplementary material for: Synchronization properties in C. elegans: Relating behavioral circuits to structural and functional neuronal connectivity
Source: PLoS Comput Biol. 2026 Sep 15;22(9):e1014152. doi: 10.1371/journal.pcbi.1014152 (PMC13588538; doi:10.1371/journal.pcbi.1014152)
Supplement: Supplementary file 1 [file pcbi.1014152.s001.pdf]

Supplementary Material

Supplementary material for *Synchronization properties in C. elegans: Relating behavioral circuits to structural and functional neuronal connectivity*

Table A. Number of shared neurons between behavioral circuits

	**For. loco.**	**Back. loco.**	**Chemo.**	**Mech.**	**Klino.**
**For. loco.**	24	0	4	4	2
**Back. loco.**	0	31	10	4	2
**Chemo.**	4	10	46	10	13
**Mech.**	4	4	10	38	0
**Klino.**	2	2	13	0	23

Table B. Rich club neurons distribution across FCs for Type I and Type II

**Type**	**FC 1**	**FC 2**	**FC 3**	**FC 4**	**FC 5**	**FC 6**
**Type I**	AVAL(R)<br>AVBL(R)<br>DVA<br>PVCL(R)	AIBR<br>RIBL	AVDL(R)<br>AVEL(R)	–	RIAR	–
**Type II**	AVAL(R)<br>PVCL(R)	AIBR<br>RIBL<br>AVBL(R)<br>DVA	AVDL(R)<br>AVEL(R)	–	RIAR	–

Table C. Comparison between the functional communities and the signaling communities

Functional Communities	Type	Signaling Communities					
		**SC 1**	**SC 2**	**SC 3**	**SC 4**	**SC 5**	**SC 6**
**FC 1**	I (II)	4 (4)	2 (1)	7 (3)	4 (4)	0 (0)	0 (0)
**FC 2**	I (II)	8 (7)	14 (13)	13 (17)	6 (5)	4 (4)	1 (1)
**FC 3**	I (II)	14 (15)	3 (5)	7 (7)	8 (9)	8 (8)	0 (0)
**FC 4**	I & II	11	11	6	6	5	1
**FC 5**	I & II	6	7	0	3	2	0
**FC 6**	I & II	4	1	1	0	1	0

Note. The table lists the number of shared neurons between the FCs from both type I and type II with the signaling communities.

Table D. Comparison between the signaling communities and the behavioral circuits

Behavioral Circuits	Signaling Communities					
	**SC 1**	**SC 2**	**SC 3**	**SC 4**	**SC 5**	**SC 6**
**Forward Locomotion**	1	0	5	2	0	0
**Backward Locomotion**	6	2	2	2	0	0
**Chemosensation**	26	5	3	6	0	0
**Mechanosensation**	9	5	4	1	7	0
**Klinotaxis**	4	4	6	7	1	0

Table E. The extended locomotion circuits

Circuit	Neurons
Forward locomotion (61)	**AS1, AS2, AS3, AS4, AS5, AS6, AS7, AS8, AS9, AS10, AS11**, AVBL, AVBR, DB1, DB2, DB3, DB4, DB5, DB6, DB7, **DD1, DD2, DD3, DD4, DD5, DD6, DVA**, PVCL, PVCR, RIBL, RIBR, **RID**, VB1, VB2, VB3, VB4, VB5, VB6, VB7, VB8, VB9, VB10, VB11, **VC1, VC2, VC3, VC4, VC5, VD1, VD2, VD3, VD4, VD5, VD6, VD7, VD8, VD9, VD10, VD11, VD12, VD13**
Backward locomotion (68)	AIBL, AIBR, **AS1, AS2, AS3, AS4, AS5, AS6, AS7, AS8, AS9, AS10, AS11**, AVAL, AVAR, AVDL, AVDR, AVEL, AVER, DA1, DA2, DA3, DA4, DA5, DA6, DA7, DA8, DA9, **DD1, DD2, DD3, DD4, DD5, DD6, DVA, RID**, RIML, RIMR, VA1, VA2, VA3, VA4, VA5, VA6, VA7, VA8, VA9, VA10, VA11, VA12, **VC1, VC2, VC3, VC4, VC5, VD1, VD2, VD3, VD4, VD5, VD6, VD7, VD8, VD9, VD10, VD11, VD12, VD13**

Table F. Functional community-wise distribution of old and new locomotion circuits across Type I and Type II functional community partitions

Type I	Forward loco.		Backward loco.		Type II	Forward loco.		Backward loco.	
	Old (24)	New (61)	Old (31)	New (68)		Old (24)	New (61)	Old (31)	New (68)
**FC 1 (75)**	22 (92%)	35 (57%)	23 (74%)	36 (53%)	**FC 1 (53)**	4	14	23 (74%)	33 (49%)
**FC 2 (67)**	2	15	3	16	**FC 2 (84)**	20 (83%)	36 (59%)	3	19
**FC 3 (52)**	0	6	5	11	**FC 3 (57)**	0	6	5	11
**FC 4 (45)**	0	2	0	2	**FC 4 (45)**	0	2	0	2
**FC 5 (27)**	0	2	0	2	**FC 5 (27)**	0	2	0	2
**FC 6 (6)**	0	1	0	1	**FC 6 (6)**	0	1	0	1

The table shows how neurons from the old and new versions of the forward and backward locomotion circuits are distributed across the identified functional communities under Type I and Type II functional community partitions.

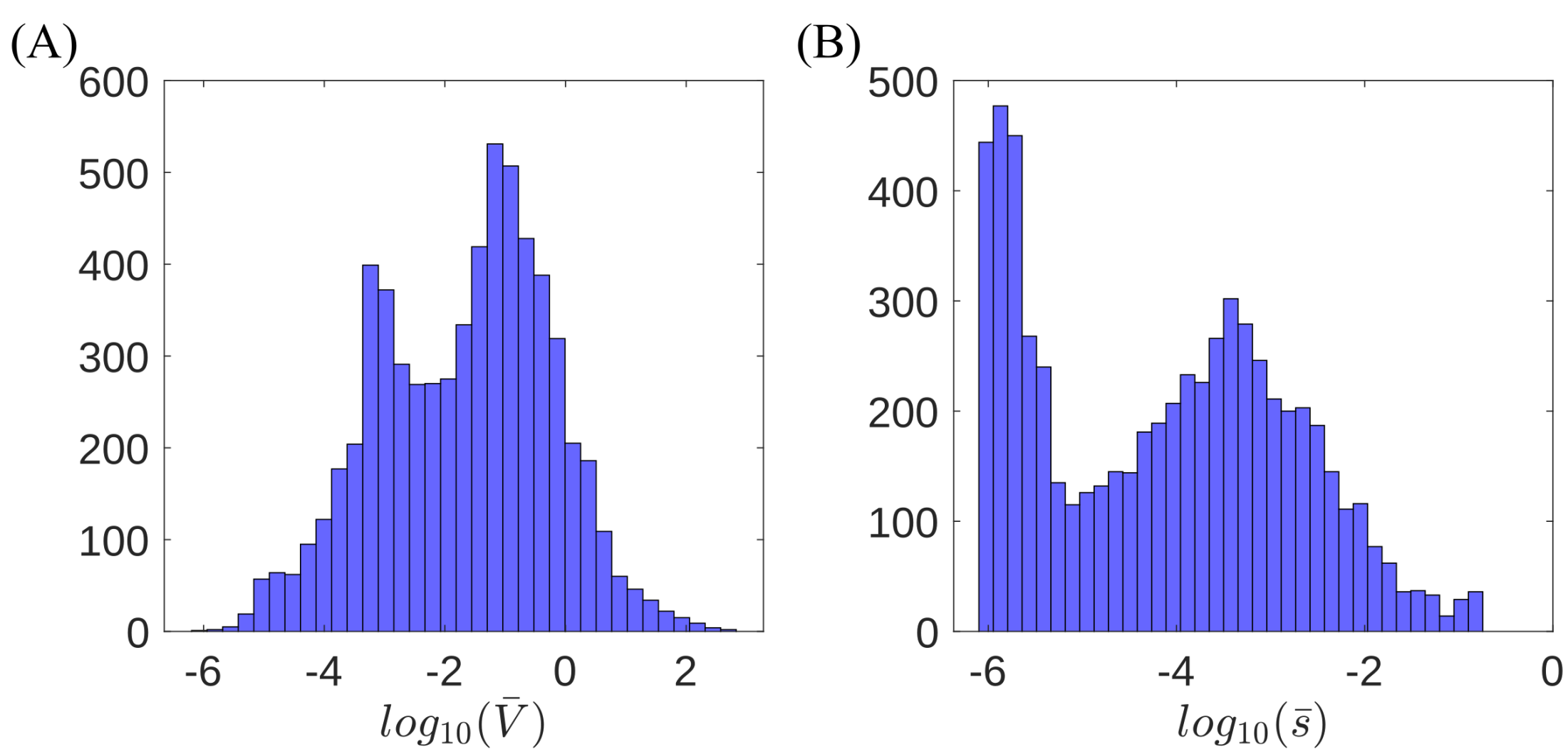

Fig A. Range of oscillation of the neuron responses. These are calculated as $\bar{V} = \max(V_i) - \min(V_i)$ and $\bar{s} = \max(s_i) - \min(s_i)$ over the last 20% time series data. (A) Histogram of the response voltage of the neuron. (B) Same for the synaptic variable. Data consist of 23 cases for $I_i^{ext} = 5 \times 10^3$ where we find oscillatory response.

Table G. Comparison of results for different datasets

**Result**	**Our weighted data (W)**	**Our binarized data (B)**	**Fenyves *et al.* 2020 data (F)**	**Comparison**
Chemical synapses	2194 weighted synaptic connections with a total of 6393 synapses	2194 binary connections	1718 binary connections	1011 common connections between our data (**W** and **B**) and Fenyves *et al.* 2020 data (**F**).
Synaptic polarity	2118 '+' and 76 '-' connections contributions to a total of 6239 '+' and 155 '-' synapses, respectively	2118 '+' and 76 '-' binary connections	1298 '+' and 420 '-' binary connections	788 common '+' and 21 common '-' connections between our data (**W** and **B**) and the Fenyves *et al.* 2020 data (**F**).
Susceptible neurons	78 unique neurons with 163 current-neuron pairs	180 unique neurons with 382 current-neurons pairs	181 unique neurons with 379 current-neuron pairs	60 common neurons between **W** and **B**. 123 common neurons between **B** and **F**.
Cooccurrence matrix	Off diagonal entries: minimum is 1 and maximum is 163 (possible range is from 0 to 163)	Off diagonal entries: minimum is 28 and maximum is 216 (possible range is from 0 to 382)	Off diagonal entries: minimum is 22 and maximum is 254 (possible range is from 0 to 379)	Pearson correlation: 0.56 between **W** and **B**. 0.79 between **B** and **F**.
Functional communities	Size of the FCs: 84, 57, 53, 45, 27, 13.	Size of the FCs: 72, 52, 52, 50, 48, 5.	Sizes of the FCs: 77, 59, 52, 48, 35, 8.	Adjusted Rand Index (see *Note 2* below): 0.3 (**W** and **B**), 0.33 (**B** and **F**).

Note 1. **W**, **B**, and **F** stand for our weighted data, our binarized data, and the Fenyves et al. 2020 data, respectively. We focus on the *binary* connectivity (both chemical synapses and gap junctions) for a direct comparison using the 277 neurons that are present in both datasets, which eliminates potentially confounding effects due to different synaptic weights in the two datasets. This also enables us to check the robustness of our results for different versions of our connectome (**W** and **B**). For the two missing neurons (PLML/R) in the Fenyves *et al.* 2020 data that are present in our data, we carry over the our connectivity data to the Fenyves *et al.* 2020 data. In this way, both datasets (**B** and **F**) have the same binarized gap junction connectivity and differ only in the binarized chemical synapse connectivity. Binarization is also viable because (i) our main conclusions are robust when binarizing our connectome data (see below), and (ii) all synapses between a pair of neurons in the prediction data sets of Fenyves et al. 2020 are of the same sign. Moreover, when using the Fenyves et al. 2020 dataset, we excluded 1,797 synaptic connections classified as complex or unpredicted because their specific identities as excitatory or inhibitory were unavailable, whereas this distinction is required for our model simulations.
Note 2. Adjusted Rand Index (ARI) ranges from -1 to 1 and is used to show the similarity between different functional community partitionings, where an ARI near 1 means the two community partitions group the exact same neurons together, a score near 0 indicates their agreement is purely random, and a negative score means the grouping is worse than random guessing.

Table H. Functional community-wise distribution of the behavioral circuits across three functional community partitionings derived from the three datasets W, B, and F which stand for our weighted data, our binarized data, and the binarized Fenyves *et al.* 2020 data, respectively.

FC	Behavioral Circuits														
	Forward loco. (24)			Backward loco. (31)			Chemosen-sation(46)			Mechanosen-sation(38)			Klinotaxis (23)		
	W	B	F	W	B	F	W	B	F	W	B	F	W	B	F
**FC 1**	4	1	1	23	18	19	6	3	3	7	4	3	1	0	0
**FC 2**	20	21	21	3	0	0	6	2	2	6	2	7	8	2	3
**FC 3**	0	0	0	5	5	4	13	9	4	14	7	7	3	4	1
**FC 4**	0	0	0	0	2	0	9	20	20	8	17	9	8	6	11
**FC 5**	0	0	0	0	0	0	6	10	2	2	1	0	2	4	0
**FC 6**	0	2	2	0	6	8	6	2	15	1	7	12	1	7	8

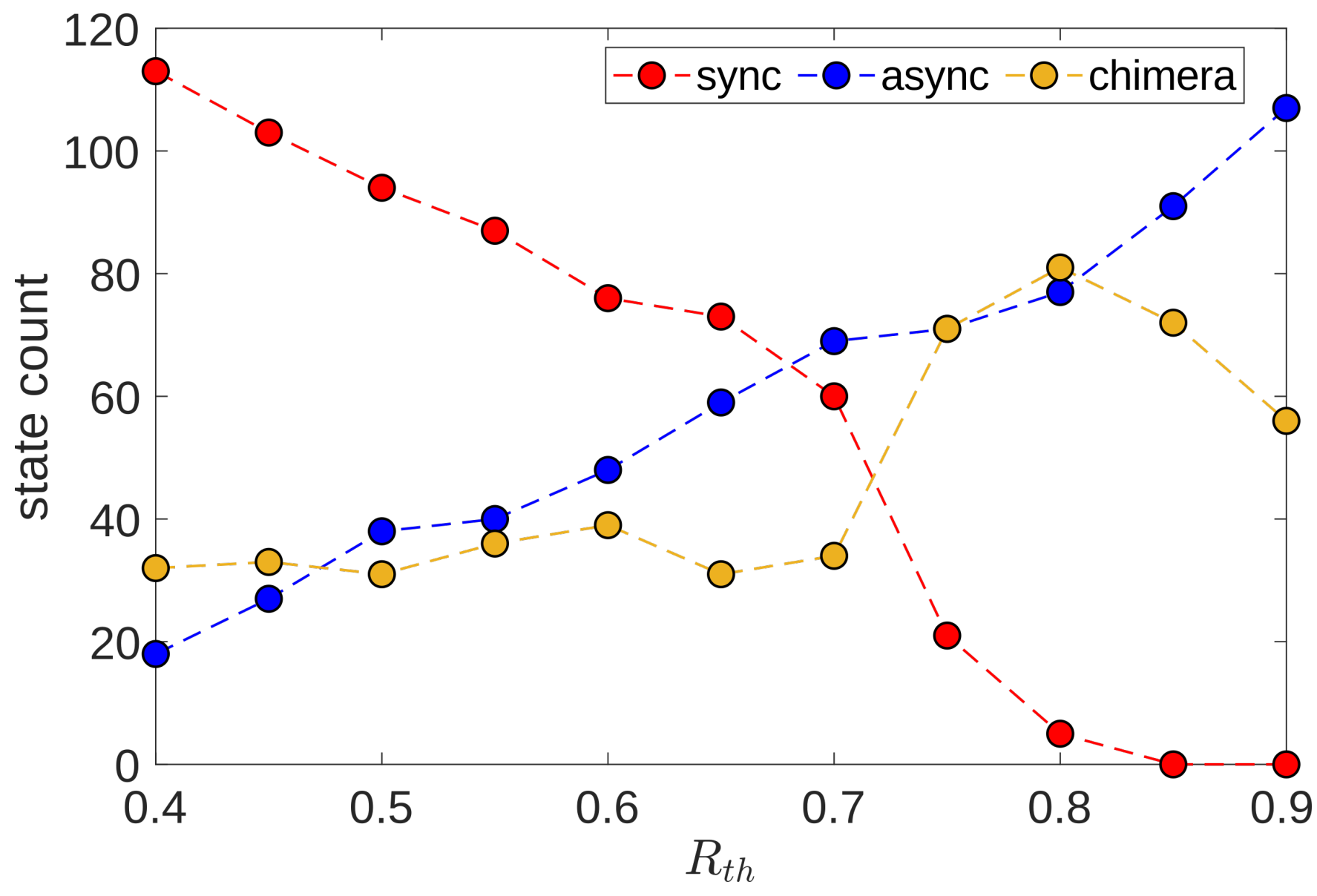

Fig B. Changes in the count of the sync, async and chimera patterns with R_{th}. Here we vary the synchronization threshold R_{th} for synchronous community detection using the Louvain algorithm. As R_{th} is increased, we see the number of sync patterns decrease and async patterns increase which is expected.

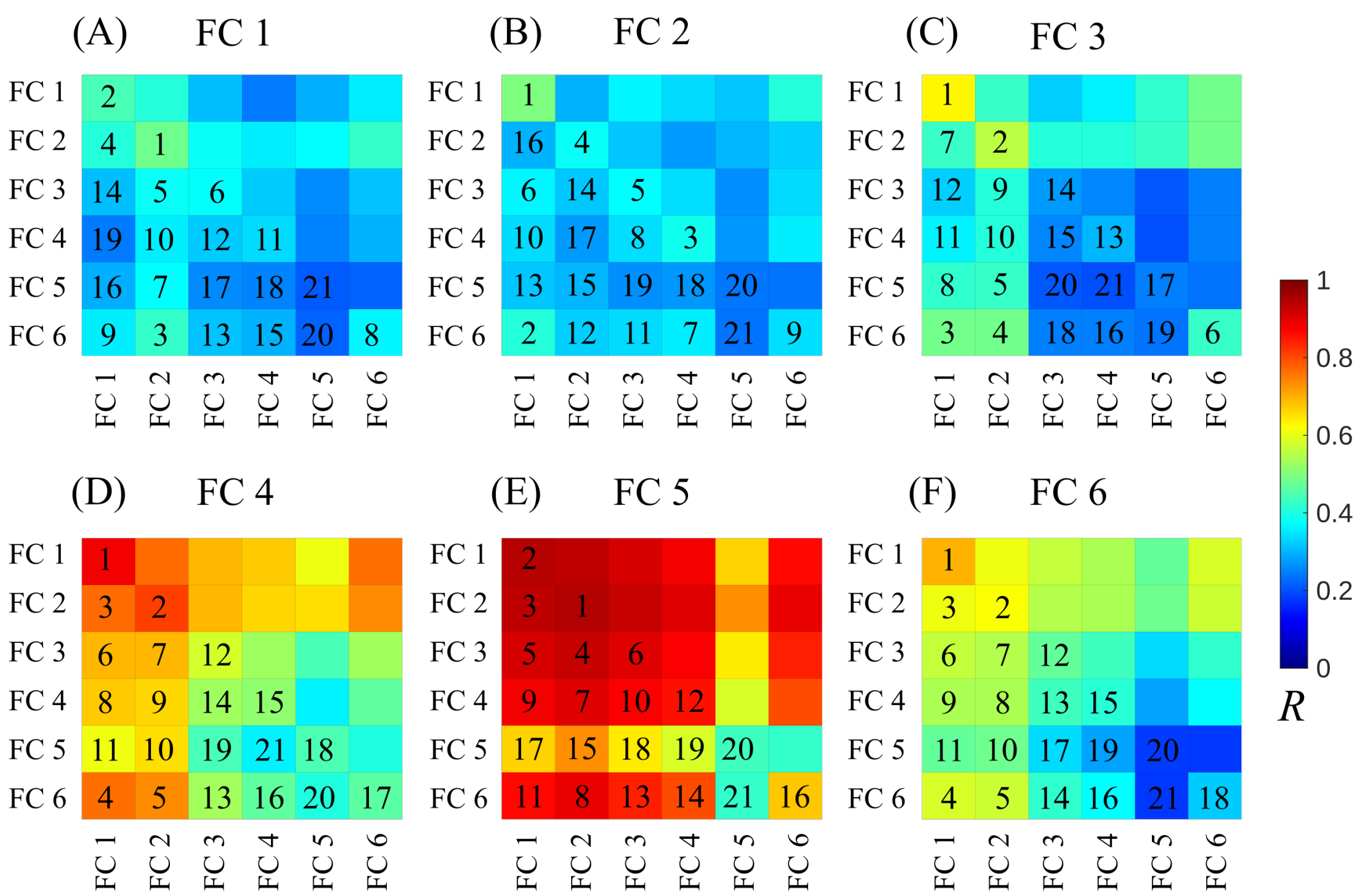

Fig C. Averaged Kuramoto order matrix R for stimulation applied to neurons in different FCs from type II. Panels (A)-(F) correspond to stimulation of neurons in FC 1 through FC 6, respectively. The colorbar indicates the value of R, reflecting the degree of synchronization between pairs of FCs. The rank numbers in each matrix are assigned in decreasing order of R, where 1 denotes the highest level of synchronization and 21 denotes the lowest.

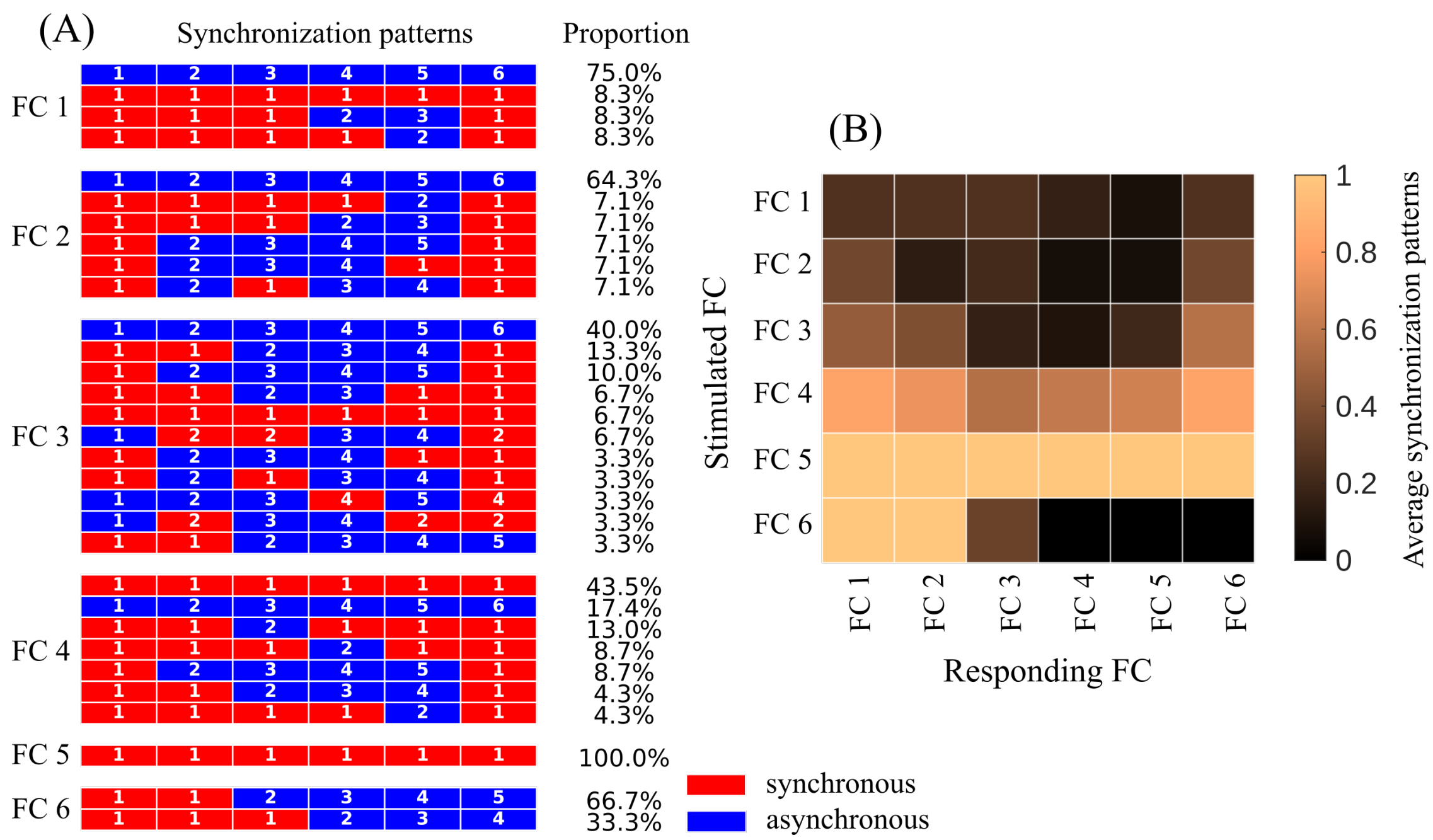

Fig D. Emergent synchronization patterns following FC-wise stimulation with FCs from WSBM type II. A synchronization threshold $R_{th} = 0.6$ is used to generate these patterns. (A) Synchronization pattern types and their proportions for each FC when its neurons are stimulated. (B) Probability of synchronization.

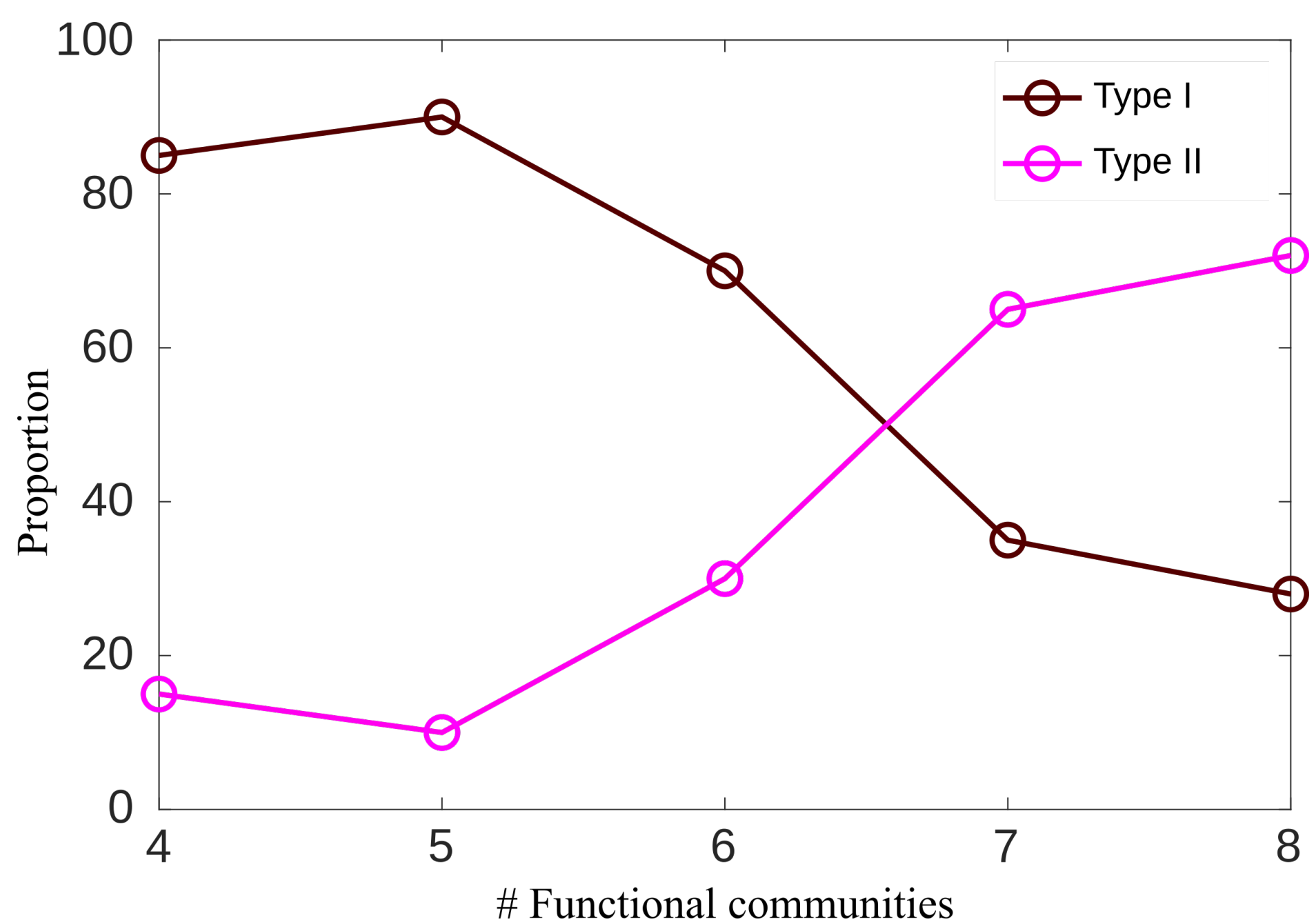

Fig E. Effect of number of communities. Change in the proportions of Type I and Type II partitions as the number of communities varies.

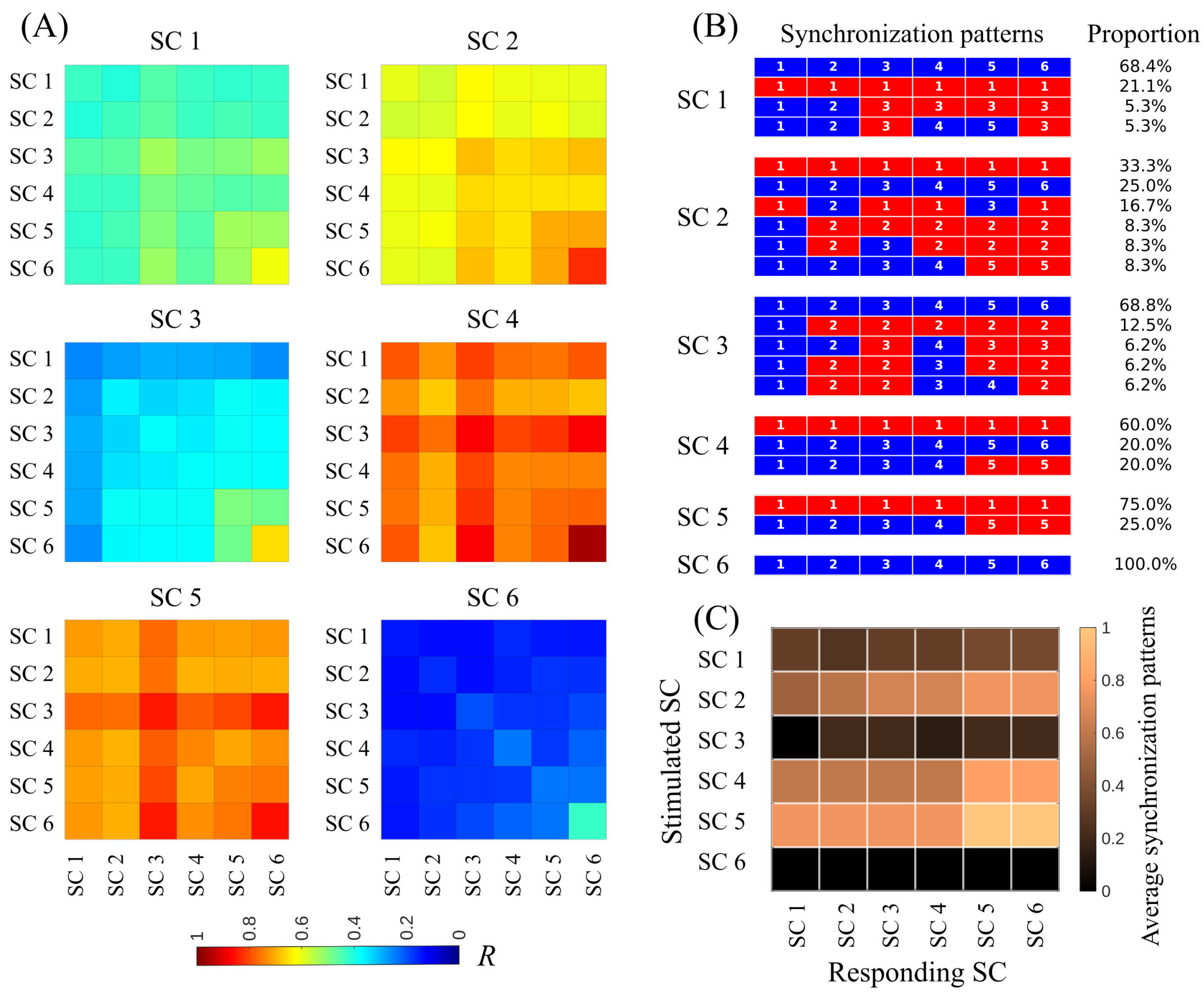

Fig F. Synchronization patterns in the signaling communities. (A) Average Kuramoto order matrix when neurons of a SC are stimulated. (B) Synchronization patterns and their respective proportions after synchronous community detection using the Louvain algorithm with $R_{th} = 0.6$. (C) Probability of a SC being a member of the synchronized group when neurons of some other SC are stimulated. Row-wise driving and column-wise response are shown. The resulting synchronization patterns appear more homogeneous across SCs than what we have observed across FCs.

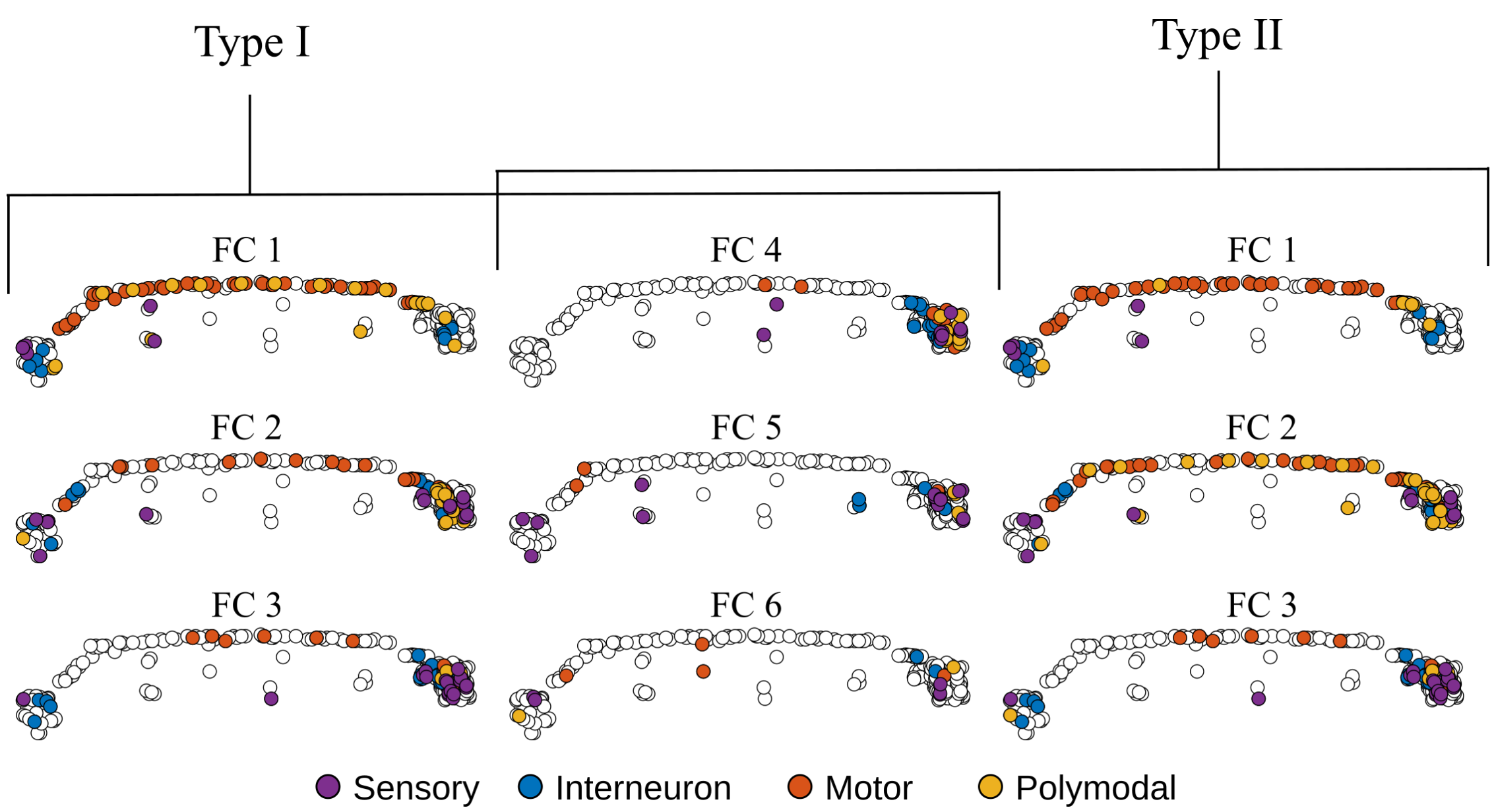

Fig G. The positional distribution of neurons in each FCs from WSBM type I and type II. The head of *C. elegans* in this figure is towards the right side of the screen from the reader's point of view.

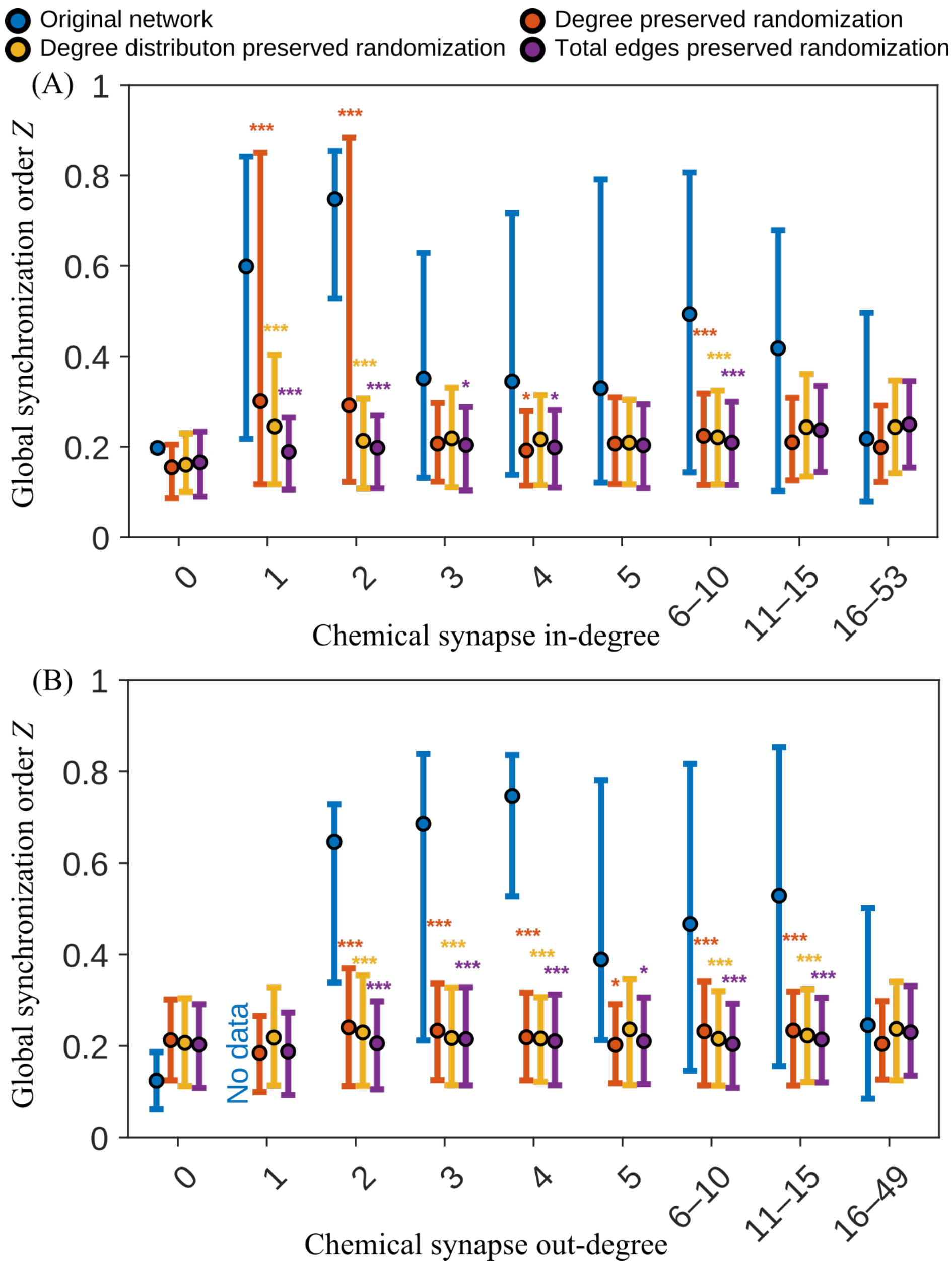

Fig H. Relation between the global synchronization order Z and chemical synapse degree of the stimulated neuron: The original network and the ensemble averages of its three randomized variants are shown, similar to Fig. 7. The dot denotes the mean value and the lower and upper edges represent the 10th and 90th percentiles, respectively. p values are used for statistical significance where *, **, and *** denote $p < 0.05$, 0.01, and 0.001, respectively. (A) Chemical synapse in-degree, (B) chemical synapse out-degree.

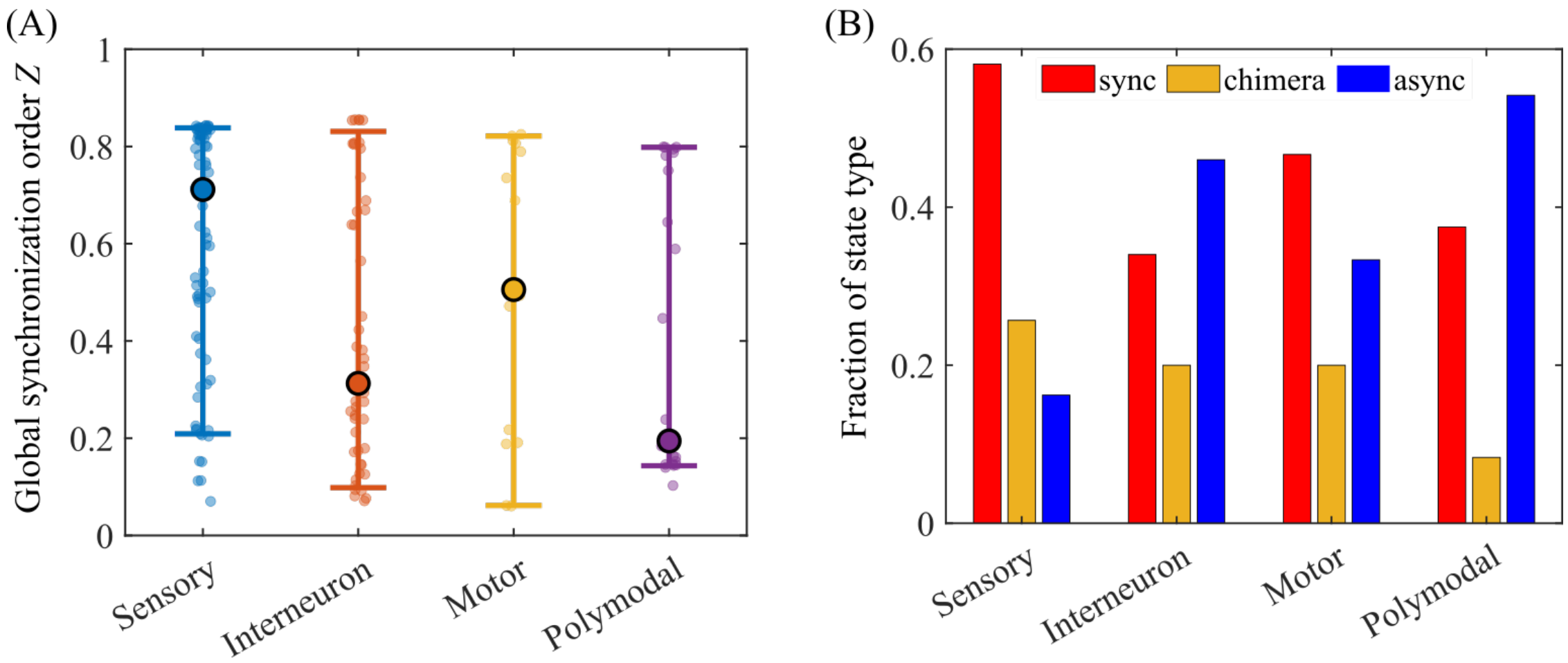

Fig I. Synchronization patterns and their relation to stimulated neuron types. (A) The global synchronization order Z is measured and grouped according to the stimulation of different neuron types. The big dots denote the median values, and the lower and upper edges represent the 10th and 90th percentiles, respectively. (B) The fraction of different synchronization patterns are plotted for each neuron type. A synchronization threshold $R_{th} = 0.6$ is used to determine these synchronization pattern using the Louvain synchronous community detection method.

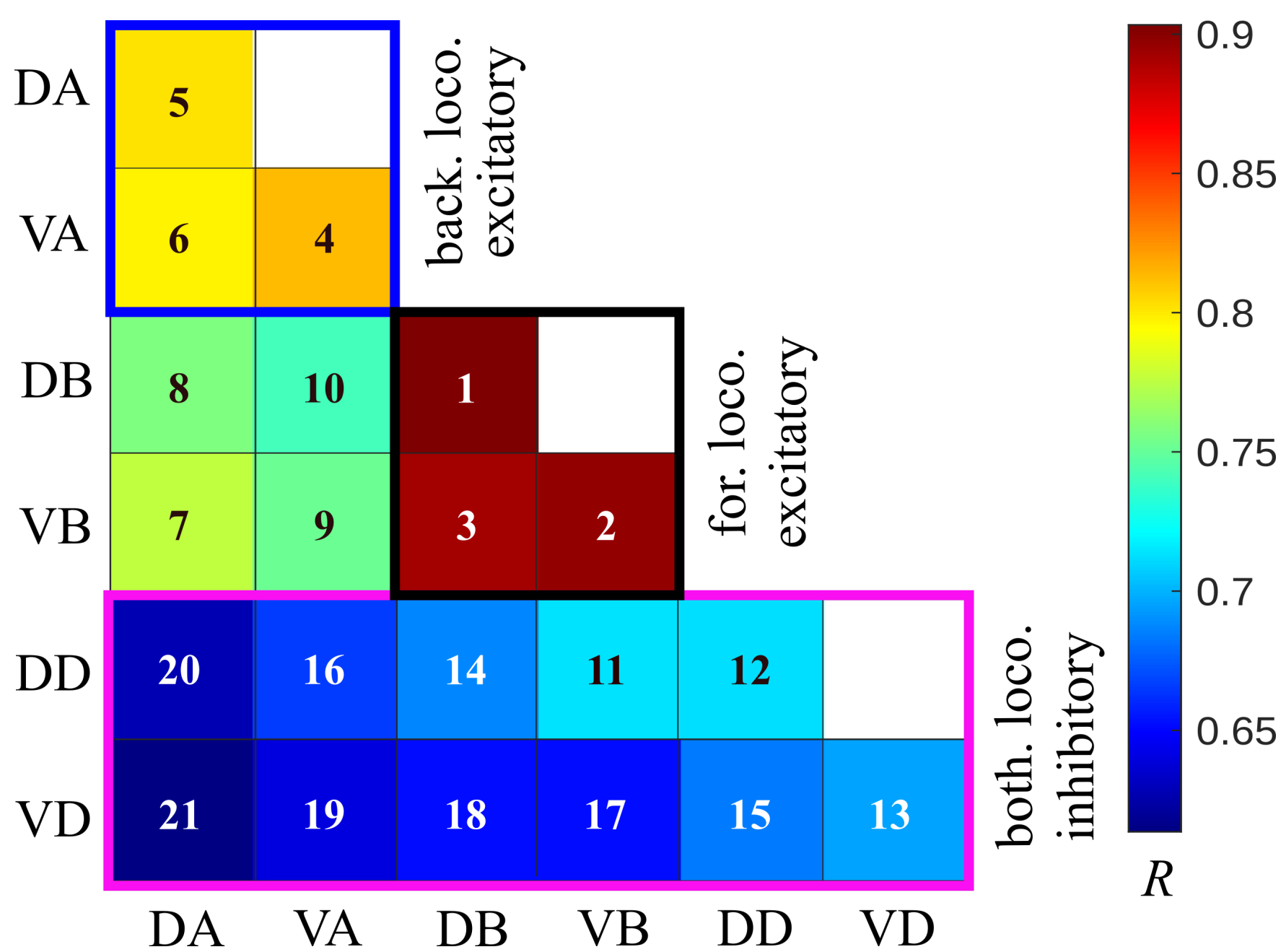

Fig J. Synchronization between the neurons from six motor neuron classes involved in locomotion. Numbers indicate rank numbers of the entries in descending order.

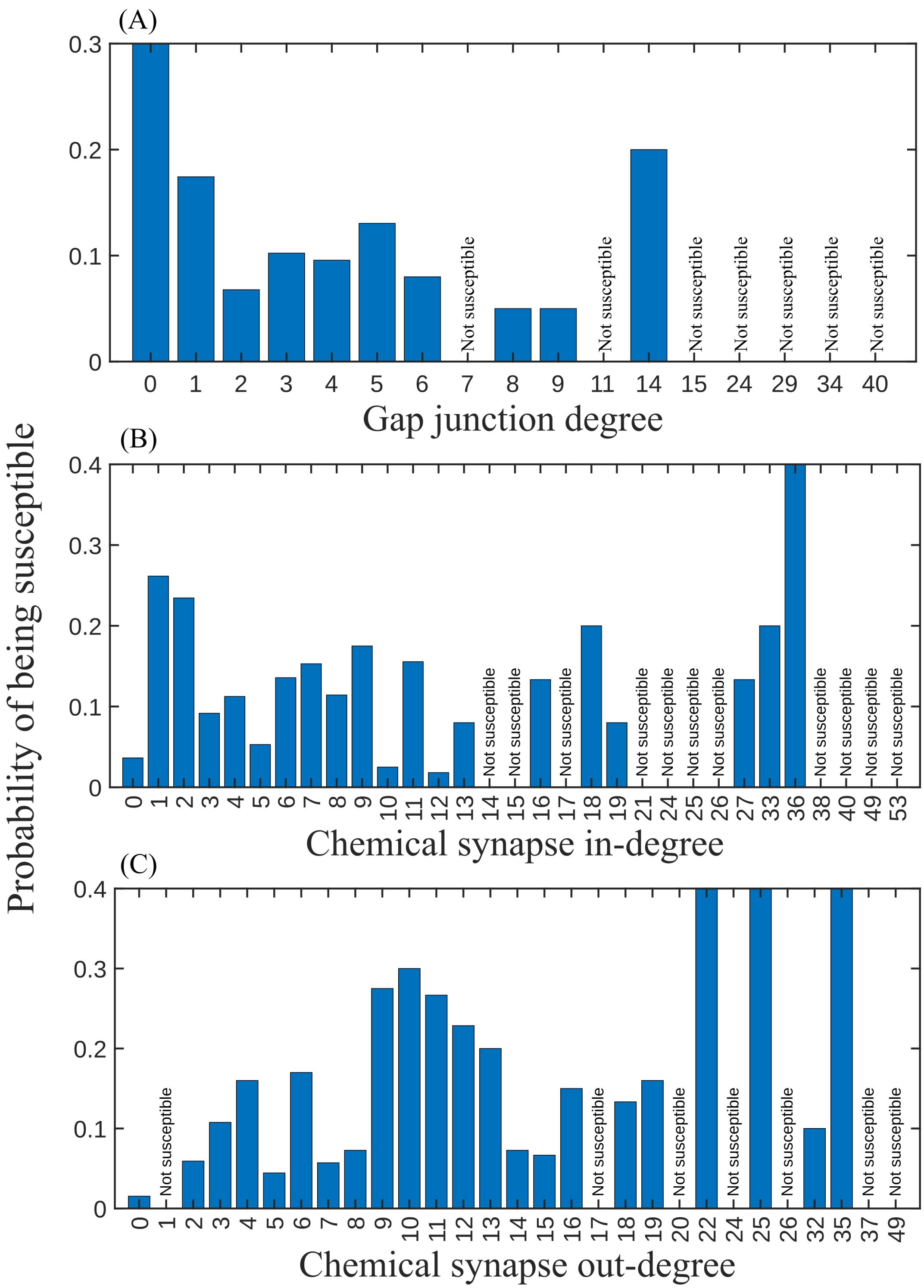

Fig K. Relation between a neuron being susceptible to stimulation and structural network properties. (A) Low gap junction degree neurons are more probable of being susceptible to stimulation than the high degree ones. There is no such relation of susceptibility with the (B) chemical synapse in-degree, and (C) chemical synapse out-degree.

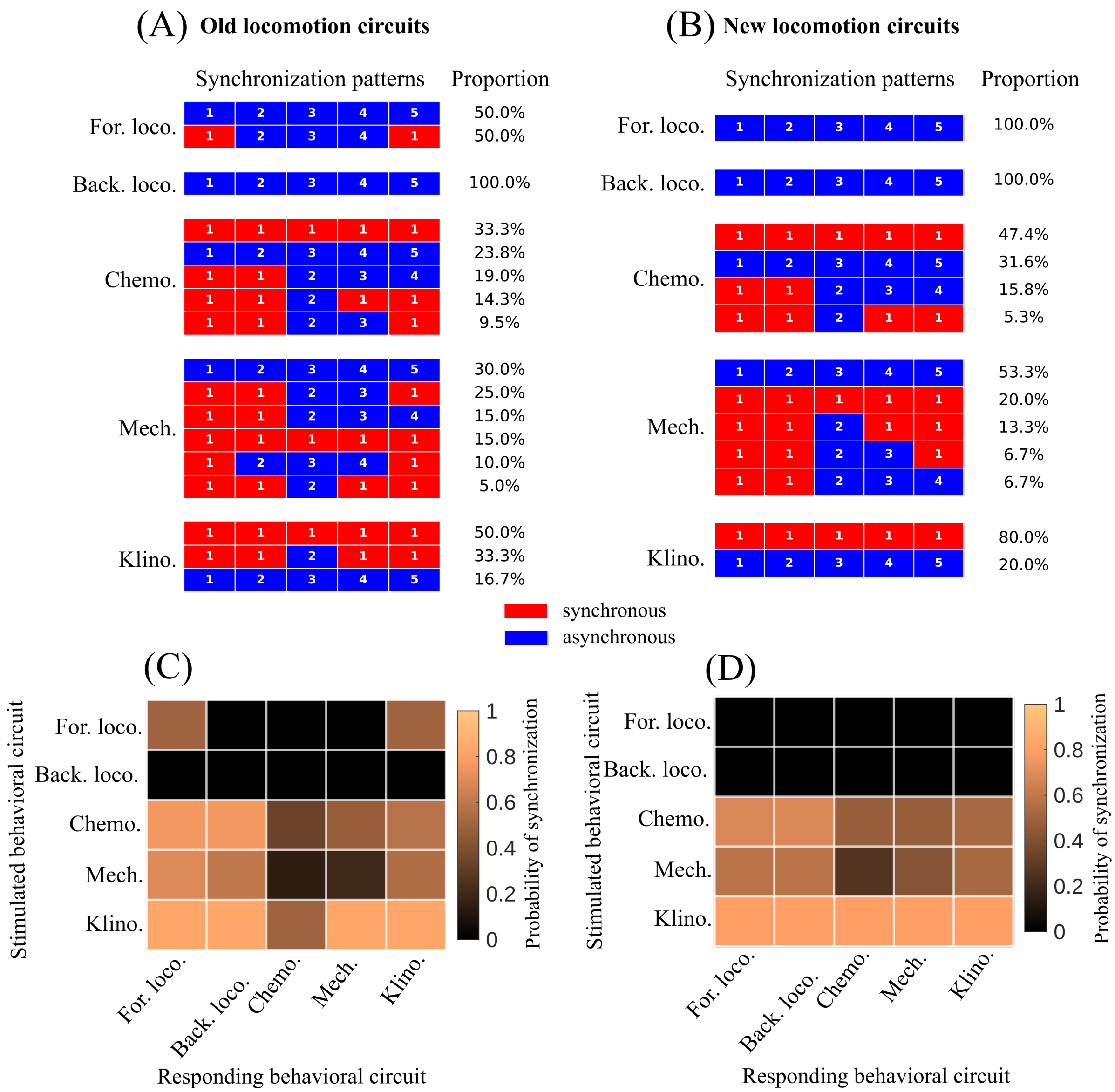

Fig L. Comparison between the synchronization patterns with old and new (extended) locomotion circuits.
