# Supplementary material for: Synchronization properties in C. elegans: Relating behavioral circuits to structural and functional neuronal connectivity
Source: PLoS Comput Biol. 2026 Sep 15;22(9):e1014152. doi: 10.1371/journal.pcbi.1014152 (PMC13588538; doi:10.1371/journal.pcbi.1014152)
Supplement: Supplementary file 2 [file pcbi.1014152.s002.pdf]

Review of "Synchronization properties in *C. elegans*: Relating behavioral circuits to structural and functional neuronal connectivity."

Overall, the authors employed an interesting set of techniques to contribute to the understanding of the *C. elegans* nervous system and the relationship between structural connectivity, functional connectivity, and behavior. They derived some interesting and novel results using computational models.

1. The authors assume that GABA neurons are inhibitory and glutamatergic and cholinergic neurons are excitatory. There is work indicating that this assumption does not hold for *C. elegans*. For example, Ref [53] (cited by the authors), finds that both the neurotransmitters released by the presynaptic neurons and the type of receptors expressed by the postsynaptic neurons in tandem determine whether the chemical synapse is excitatory or inhibitory.

   (a) To what extent do the signs used by the authors align with sign estimates currently available?
   (b) To what extent are the findings in this manuscript dependent on the synaptic signs assumed by the authors? Would the authors achieve the same results using the signs predicted by Ref [53]? It is difficult to judge the reliability of the results when it is unclear how robust the results are to different assumptions.

   The following is an incomplete list of publications that contain sign predictions using a variety of techniques:

Lanza, E., Di Angelantonio, S., Gosti, G., Ruocco, G., & Folli, V. (2021). A recurrent neural network model of C. elegans responses to aversive stimuli. *Neurocomputing*, *430*, 1-13.

Piggott, B. J., Liu, J., Feng, Z., Wescott, S. A., & Xu, X. S. (2011). The neural circuits and synaptic mechanisms underlying motor initiation in C. elegans. *Cell*, *147*(4), 922-933.

Rakowski, F., Srinivasan, J., Sternberg, P. W., & Karbowski, J. (2013). Synaptic polarity of the interneuron circuit controlling C. elegans locomotion. *Frontiers in computational neuroscience*, *7*, 128.

Fenyves, B. G., Szilágyi, G. S., Vassy, Z., Sőti, C., & Csermely, P. (2020). Synaptic polarity and sign-balance prediction using gene expression data in the Caenorhabditis elegans chemical synapse neuronal connectome network. *PLOS Computational Biology*, *16*(12), e1007974.

2. In the example shown in Figure 3, the voltage level of the neurons in (c) appear to be many orders of magnitude higher than what tends to be measured from neurons. I would expect the voltage levels to fall between -120 and 100 mV. Spiking neuron (which these are not) achieve a peak voltage when spiking of 50 mV. Why are some of the neurons showing activity levels in the 5000 to 15000 mV range and is this realistic? Is there evidence that these neurons achieve these high voltage levels when stimulated with input current? If the activity traces of the simulated neurons are not realistic how does this impact the computations done on these traces, the results, and the conclusions?

3. As a follow up to the previous point, the authors point out that the membrane potentials of neurons vary in range and amplitude and so they normalize them to the interval [-1, 1]. However, the amplitudes of the oscillations appear to be negligible compared to the mid-range values achieved by the neurons (e.g. Fig. 3(c)) . With this normalization the mid-range value is essentially thrown out. Why is the oscillation kept as relevant information, but the mid-range value is not considered to be important?

4. Are the original voltage traces or the normalized voltage traces used to compute the correlation based functional community detection?

5. It is my understanding that the gap junctions were found to be responsible for the functional connectivity groups. What role, if any, do the synapses play in determining the functional connectivity? Are they irrelevant?

6. How do the authors' results fit with previous findings in the literature? A more specific and direct comparison would be helpful.

7. The code used to generate the results should be made publicly available according to the policies of PLOS Computational Biology.
