# Supplementary material for: Synchronization properties in C. elegans: Relating behavioral circuits to structural and functional neuronal connectivity
Source: PLoS Comput Biol. 2026 Sep 15;22(9):e1014152. doi: 10.1371/journal.pcbi.1014152 (PMC13588538; doi:10.1371/journal.pcbi.1014152)
Supplement: Supplementary file 3 [file pcbi.1014152.s003.pdf]

Response to Reviewers

Synchronization properties in *C. elegans*: Relating behavioral circuits to structural and functional neuronal connectivity

Gourab Kumar Sar, Andrew Patton, Emma Towlson, and Jörn Davidsen

Manuscript ID: PCOMPBIOL-D-26-00649

Response to Reviewer 1

Comment: Overall, the authors employed an interesting set of techniques to contribute to the understanding of the C. elegans nervous system and the relationship between structural connectivity, functional connectivity, and behavior. They derived some interesting and novel results using computational models.

Response: We thank the reviewer for the positive evaluation of our work. Below we provide a point-by-point response to each of the comments raised by the reviewer.

Comment 1: The authors assume that GABA neurons are inhibitory and glutamatergic and cholinergic neurons are excitatory. There is work indicating that this assumption does not hold for C. elegans. For example, Ref [53] (cited by the authors), finds that both the neurotransmitters released by the presynaptic neurons and the type of receptors expressed by the postsynaptic neurons in tandem determine whether the chemical synapse is excitatory or inhibitory.
(a) To what extent do the signs used by the authors align with sign estimates currently available?
(b) To what extent are the findings in this manuscript dependent on the synaptic signs assumed by the authors? Would the authors achieve the same results using the signs predicted by Ref [53]? It is difficult to judge the reliability of the results when it is unclear how robust the results are to different assumptions.
The following is an incomplete list of publications that contain sign predictions using a variety of techniques:
Lanza, E., Di Angelantonio, S., Gosti, G., Ruocco, G., & Folli, V. (2021). A recurrent neural network model of C. elegans responses to aversive stimuli. Neurocomputing, 430, 1-13.
Piggott, B. J., Liu, J., Feng, Z., Wescott, S. A., & Xu, X. S. (2011). The neural circuits and synaptic mechanisms underlying motor initiation in C. elegans. Cell, 147(4), 922-933.
Rakowski, F., Srinivasan, J., Sternberg, P. W., & Karbowski, J. (2013). Synaptic polarity of the interneuron circuit controlling C. elegans locomotion. Frontiers in computational neuroscience, 7, 128.
Fenyves, B. G., Szilágyi, G. S., Vassy, Z., Sőti, C., & Csermely, P. (2020). Synaptic polarity and sign-balance prediction using gene expression data in the Caenorhabditis elegans chemical synapse neuronal connectome network. PLOS Computational Biology, 16(12), e1007974.

Response: Thanks for this insightful comment. It is indeed true that the polarity (excitatory/inhibitory) of a chemical synapse is determined by both the neurotransmitter released by the presynaptic neuron and type of receptor expressed by the postsynaptic neuron. However, experimental determination of the synaptic polarity is more challenging than inferring the direction of the synapse, and has not yet been achieved at the full connectome level. Having described this difficulty, Fenyves *et al.* [1] — the last reference listed by the reviewer and Ref [53] of our original manuscript — tried to predict the synaptic polarity using the connectome data and manually curated gene expression data for all the synapses in *C. elegans* chemical network. To answer the

concerns the reviewer raised in this comment, we carry out our analyses using this sign prediction data (with certain modifications which were required for comparison with our dataset as discussed below).

Response to (a): Upon comparing the synaptic signs in our dataset and the prediction dataset in Fenyves *et al.* [1], we found that the number of (unweighted) connections where an unambiguous sign description (excitatory or inhibitory) is available in both datasets is 999. Out of these, 797 (around 80%) have the same sign in the two datasets. 201 connections are considered excitatory in our dataset and are categorized as inhibitory in prediction data in Fenyves *et al.* [1]. There is a single connection which is inhibitory in our data, and predicted as excitatory by Fenyves *et al.*. It is important to note here that the study of Fenyves *et al.* [1] predicted a significant number of synapses as *complex* (both excitatory and inhibitory) and *unpredicted*. Between the 277 common neurons used in both the datasets, 1797 out of the 3503 connections in Fenyves *et al.* [1] (more than 50%) among these neurons are categorized as complex or unpredicted by Fenyves *et al.*.

Response to (b): Before diving into the comparative analysis of the synchronization behavior of the two data sets with different chemical synaptic signs, we would like to emphasize first that one of the main findings of our initial study was that (undirected) gap junctions play a more significant role compared to the chemical synapses although the number of total gap junctions (890) is much smaller than the number of total chemical synapses (6393). Hence, it is expected that our results remain largely unchanged despite the differences in synaptic polarity. This is indeed what we find.

To isolate the effect of changes in synaptic polarity, we decided to focus on the *binary* connectivity (both chemical synapses and gap junctions) for a direct comparison using the 277 neurons that are present in both datasets, which eliminates potentially confounding effects due to different synaptic weights in the two datasets. For the two missing neurons (PLML/R) in the Fenyves *et al.* 2020 data that are present in our data, we carry over the our connectivity data to the Fenyves *et al.* 2020 data. In this way, both datasets have the same binarized gap junction connectivity and differ only in the binarized chemical synapse connectivity. Binarization is also viable because (i) our main conclusions are robust when binarizing our connectome data (see below), and (ii) all synapses between a pair of neurons in the prediction data sets of Fenyves *et al.* are of the same sign. Moreover, when using the Fenyves et al. dataset, we excluded 1,797 synaptic connections classified as complex or unpredicted because their specific identities as excitatory or inhibitory were unavailable, whereas this distinction is required for our model simulations.

The comparison is presented in Table 1, which also includes the results with the weighted connectivity data used in our original study. As the connections and their signs vary across the datasets, we observe variability in neuronal dynamics (for example, which neurons are susceptible upon stimulation changes with the dataset used). However, the collective neuronal dynamics remain largely unaltered, and our conclusions remain valid:

- In both functional community partitioning derived from the the binarized version of our data and Fenyves *et al.* data, the forward and backward locomotion circuits remain segregated (roughly more than 60% neurons belong to only one of the FCs). This is in line with our first conclusion that locomotion circuits function largely independently.

- The sensory circuits (chemosensation, mechanosensation, and klinotaxis) remain functionally distributed across the FCs. No more than 45% of neurons in any of these circuits belong to a single single FC. This is again consistent with our second conclusion that sensory circuits comprise multiple functional components.

In Table 2, we compare the distribution of the neurons in the behavioral circuits across the functional communities achieved using the different datasets. In summary, the main claims of our study remain

valid even when we consider changes in synaptic polarity as proposed in [1]. There are expected variations at the micro scale, which do not affect the collective dynamics however.

We add this discussion in our revised manuscript after **line 128**. **"Apart from this commonly used convention, recent *in silico* studies have predicted the polarity (excitatory/inhibitory) of the synapses depending on the gene expression data of the presynaptic neurotransmitters and postsynaptic receptors [58-61] (we establish the robustness of our findings using the synaptic polarity in the Discussion section.)"**

After **line 768** we add that, **"In addition, we derive FCs using this new dataset that assigns synaptic polarity, excitatory or inhibitory, at the level of individual synapses rather than defining polarity solely based on the presynaptic neuron. We report that the locomotion circuits remain segregated and the sensory circuits remain distributed across these new FCs (see Table H in the Supplementary Material). In other words, the functional organization of the behavioral circuits remain robust even when we consider the polarity of the synapses."**

Table 1: Comparison of results for different datasets

**Result**	**Our weighted data (W)**	**Our binarized data (B)**	**Fenyves *et al.* 2020 data (F)**	**Comparison**
Chemical synapses	2194 weighted synaptic connections with a total of 6393 synapses	2194 binary connections	1718 binary connections	1011 common connections between our data (**W** and **B**) and Fenyves *et al.* 2020 data (**F**).
Synaptic polarity	2118 '+' and 76 '-' connections contributions to a total of 6239 '+' and 155 '-' synapses, respectively	2118 '+' and 76 '-' binary connections	1298 '+' and 420 '-' binary connections	788 common '+' and 21 common '-' connections between our data (**W** and **B**) and the Fenyves *et al.* 2020 data (**F**).
Susceptible neurons	78 unique neurons with 163 current-neuron pairs	180 unique neurons with 382 current-neurons pairs	181 unique neurons with 379 current-neuron pairs	60 common neurons between **W** and **B**. 123 common neurons between **B** and **F**.
Cooccurrence matrix	Off diagonal entries: minimum is 1 and maximum is 163 (possible range is from 0 to 163)	Off diagonal entries: minimum is 28 and maximum is 216 (possible range is from 0 to 382)	Off diagonal entries: minimum is 22 and maximum is 254 (possible range is from 0 to 379)	Pearson correlation: 0.56 between **W** and **B**. 0.79 between **B** and **F**.
Functional communities	Size of the FCs: 84, 57, 53, 45, 27, 13.	Size of the FCs: 72, 52, 52, 50, 48, 5.	Sizes of the FCs: 77, 59, 52, 48, 35, 8.	Adjusted Rand Index (see *Note 2* below): 0.3 (**W** and **B**), 0.33 (**B** and **F**).

Note 1. **W**, **B**, and **F** stand for our weighted data, our binarized data, and the Fenyves et al. 2020 data, respectively. We focus on the *binary* connectivity (both chemical synapses and gap junctions) for a direct comparison using the 277 neurons that are present in both datasets, which eliminates potentially confounding effects due to different synaptic weights in the two datasets. This also enables us to check the robustness of our results for different versions of our connectome (**W** and **B**). For the two missing neurons (PLML/R) in the Fenyves *et al.* 2020 data that are present in our data, we carry over the our connectivity data to the Fenyves *et al.* 2020 data. In this way, both datasets (**B** and **F**) have the same binarized gap junction connectivity and differ only in the binarized chemical synapse connectivity. Binarization is also viable because (i) our main conclusions are robust when binarizing our connectome data (see below), and (ii) all synapses between a pair of neurons in the prediction data sets of Fenyves et al. 2020 are of the same sign. Moreover, when using the Fenyves et al. 2020 dataset, we excluded 1,797 synaptic connections classified as complex or unpredicted because their specific identities as excitatory or inhibitory were unavailable, whereas this distinction is required for our model simulations.
Note 2. Adjusted Rand Index (ARI) ranges from -1 to 1 and is used to show the similarity between different functional community partitionings, where an ARI near 1 means the two community partitions group the exact same neurons together, a score near 0 indicates their agreement is purely random, and a negative score means the grouping is worse than random guessing.

Table 2: Functional community-wise distribution of the behavioral circuits across three functional community partitionings derived from the three datasets **W**, **B**, and **F** which stand for our weighted data, our binarized data, and the binarized Fenyves *et al.* 2020 data, respectively.

FC	Behavioral Circuits														
	Forward loco. (24)			Backward loco. (31)			Chemosensation(46)			Mechanosensation(38)			Klinotaxis (23)		
	**W**	**B**	**F**	**W**	**B**	**F**	**W**	**B**	**F**	**W**	**B**	**F**	**W**	**B**	**F**
**FC 1**	4	1	1	23	18	19	6	3	3	7	4	3	1	0	0
**FC 2**	20	21	21	3	0	0	6	2	2	6	2	7	8	2	3
**FC 3**	0	0	0	5	5	4	13	9	4	14	7	7	3	4	1
**FC 4**	0	0	0	0	2	0	9	20	20	8	17	9	8	6	11
**FC 5**	0	0	0	0	0	0	6	10	2	2	1	0	2	4	0
**FC 6**	0	2	2	0	6	8	6	2	15	1	7	12	1	7	8

Comment 2: In the example shown in Figure 3, the voltage level of the neurons in (c) appear to be many orders of magnitude higher than what tends to be measured from neurons. I would expect the voltage levels to fall between -120 and 100 mV. Spiking neuron (which these are not) achieve a peak voltage when spiking of 50 mV. Why are some of the neurons showing activity levels in the 5000 to 15000 mV range and is this realistic? Is there evidence that these neurons achieve these high voltage levels when stimulated with input current? If the activity traces of the simulated neurons are not realistic how does this impact the computations done on these traces, the results, and the conclusions?

Response: We thank the reviewer for raising this important point. We agree that the traces in the original Fig. 3(c), when plotted as raw voltage variables and labeled in mV, could be misleading. Values in the range of 5000–15000 mV are not physiologically realistic for *C. elegans* neurons, and to our knowledge there is no experimental evidence that these neurons reach such voltage levels under current stimulation.

The large numerical values in Fig. 3(c) arise from the scaling of the reduced single-compartment model used in this study. In the model, the membrane potential evolves according to

$$C\dot{V}_i = -g_l(V_i - V_{\text{cell}}) - g_e \sum_j A^{\text{gap}}_{ji}(V_i - V_j) - g_c \sum_j A^{\text{chem}}_{ji} s_j (V_i - V^s_j) + I^{\text{ext}}_i.$$

The externally applied input I^{ext}_i was used as a current-like control parameter to move the connectome-constrained dynamical system into different response regimes, including stable equilibria, new fixed points, and limit-cycle oscillations. Because the leak conductance is small relative to the numerical values of the external input, the resulting voltage variable can take very large values if interpreted literally in mV. Thus, the traces shown in the original Fig. 3(c) should not be interpreted as quantitatively realistic membrane-voltage recordings.

Following the interpretation used in the Kunert–Shlizerman–Kutz model framework [2], the relevant dynamical activity is the deviation from the equilibrium state rather than the absolute

voltage offset. We have therefore revised Fig. 3(c) to show $\Delta V_i(t) = V_i(t) - V_i^*$, where V_i^* is the equilibrium voltage of neuron i under the same stimulation condition. This removes the large equilibrium offset and displays the oscillatory activity around equilibrium, which is the quantity relevant for visualizing the limit-cycle response. After this correction, the voltage deviations shown in the revised Fig. 3(c) lie approximately in the range -250 to 250 mV (see Fig. 1 below). This range is much closer to the biologically plausible scale pointed out by the reviewer. This is also consistent with the use of the model as a reduced dynamical system and with the convention used in earlier studies based on this model [2, 3, 4].

This revision does not affect the main computations or conclusions. The functional connectivity is computed from Pearson correlations between voltage traces, and Pearson correlation is invariant under subtracting a constant offset from each trace. Therefore, replacing $V_i(t)$ by $\Delta V_i(t) = V_i(t) - V_i^*$ leaves the correlation-based functional connectivity unchanged. In addition, the phase-based synchronization analysis is performed after normalizing each neuron's voltage and synaptic activity variables to $[-1, 1]$. Thus, the Kuramoto order parameters and the resulting synchronization classifications are not affected by removing the equilibrium offset. The revision changes the visualization and clarifies the interpretation of the model output, but it does not alter the reported functional communities, synchronization patterns, or conclusions.

To clarify this point, in **line 282**, we mention that **"Following previous studies that employed the same model [21,34,35], we use the deviations of the membrane voltages from their equilibrium values (ΔV_i) to represent the relevant dynamical activity, rather than the absolute voltages."**

To further elucidate this point, in our Discussion at **line 972** we add the following: **"Thus, in our model, the external current should be interpreted primarily as a control parameter that drives the system into different dynamical regimes, rather than as a direct experimentally realistic current-injection protocol."**

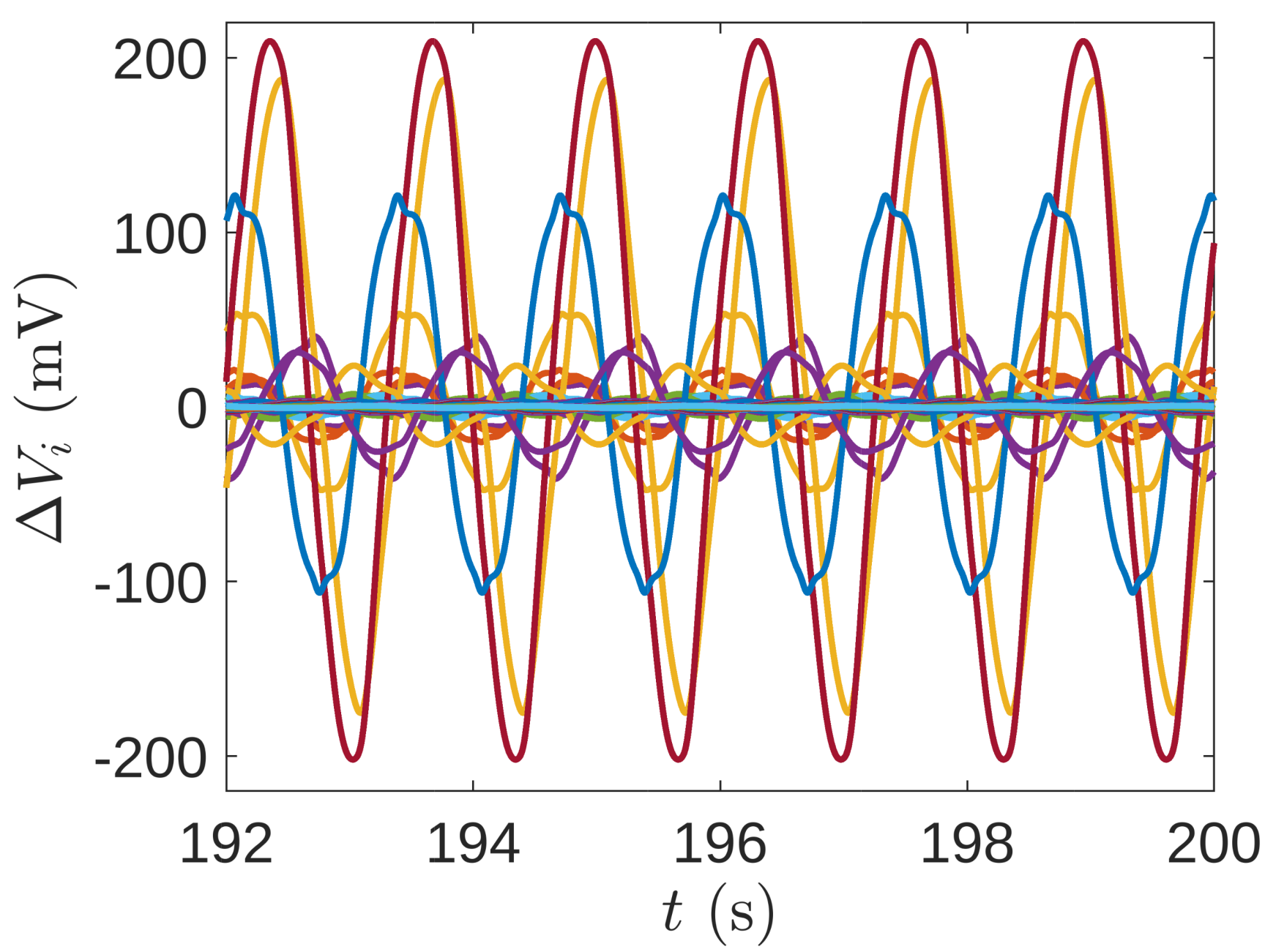

Figure 1: Voltage displacement from equilibrium.

Comment 3: As a follow up to the previous point, the authors point out that the membrane potentials of neurons vary in range and amplitude and so they normalize them to the interval [-1, 1]. However, the amplitudes of the oscillations appear to be negligible compared to the mid-range values achieved by the neurons (e.g. Fig. 3(c)). With this normalization the mid-range value is essentially thrown out. Why is the oscillation kept as relevant information, but the mid-range value is not considered to be important?

Response: In this study, we are interested in whether neurons oscillate with similar phase relationships once the system has entered a limit-cycle regime. For such a question, the absolute equilibrium offset is not the relevant quantity; rather, the relevant information is the temporal variation around the equilibrium state. This is why we normalize each neuron's voltage and synaptic activity traces before computing the phase.

We agree that, when the raw voltage variable $V_i(t)$ is plotted, the oscillatory component may appear small relative to the large mid-range value. This is precisely why the original visualization was potentially misleading. As discussed in response to the previous comment, the large mid-range value primarily reflects the equilibrium offset induced by the scaling of the reduced model and the applied current-like input. The mid-range value is not assumed to be dynamically irrelevant in general. It is important for determining the operating point of the model, the equilibrium state, and whether a given stimulation drives the system to a stable equilibrium, a new fixed point, or a limit cycle. However, once a limit-cycle response is obtained, the mid-range value acts as an offset for the oscillatory trace. Including this offset in the phase calculation would obscure the relative timing of the oscillations and would make comparisons across neurons dominated by baseline differences rather than by their oscillatory coordination.

At **line 294** we clarify this by stating - **"The mid-range subtraction removes the model-dependent equilibrium offset before phase extraction. While this offset is important for determining the operating point and dynamical regime of the system, our synchronization analysis focuses on the relative timing of oscillations around equilibrium once a limit cycle is reached. Thus, the normalization procedure allows phase relationships to be compared across neurons with different voltage offsets and amplitudes."**

Comment 4: Are the original voltage traces or the normalized voltage traces used to compute the correlation based functional community detection?

Response: For the correlation-based functional community detection reported in the manuscript, we used the original voltage traces. However, the result is identical if we decide to use the normalized voltage traces since Pearson correlation is invariant under subtracting a constant offset and multiplying each signal by a positive scale factor. Therefore, whether one uses the original voltage, the equilibrium-subtracted trace, or the normalized trace, the resulting pairwise correlation matrix remains unchanged up to numerical precision.

We have added this point in our revised manuscript. At **line 330 after Eq. 8** we add that, **"Because Pearson correlation is invariant under subtracting a constant offset and rescaling each individual trace by a positive constant, the same correlation matrix is obtained whether one uses the original voltage traces, the equilibrium-subtracted traces, or the normalized voltage traces."**

Comment 5: It is my understanding that the gap junctions were found to be responsible for the functional connectivity groups. What role, if any, do the synapses play in determining the functional connectivity? Are they irrelevant?

Response: The reviewer is correct that gap junctions have a dominant effect on the formation

of the functional communities. However, chemical synapses are not irrelevant. Their role is more fundamental in generating the neuronal activity itself. In our model, chemical synapses provide the nonlinear coupling through the synaptic activity variables s_i, and this nonlinear feedback is essential for producing oscillatory responses. In the absence of chemical synapses, the system does not exhibit the same stimulation-induced oscillatory activity on which our functional connectivity and synchronization analyses are based. Indeed, we numerically evaluated the eigenvalues of the system in the absence of chemical synapses and found that all eigenvalues have negative real parts, indicating that the equilibrium is linearly stable and that the system always converges to it. Thus, gap junctions primarily shape the correlation-based functional community structure, whereas chemical synapses are essential for enabling the dynamical responses from which these communities are inferred.

To highlight this point, we have added the following statement in **line 761** of the revised manuscript: **"While gap junctions primarily shape the correlation-based functional community structure through diffusive voltage coupling, chemical synapses provide the synaptic feedback required for the stimulation-induced oscillatory responses from which functional connectivity is inferred."**

Comment 6: How do the authors' results fit with previous findings in the literature? A more specific and direct comparison would be helpful.

Response: We thank the reviewer for this helpful suggestion. We have now added a more explicit comparison with previous studies in the Discussion section (under "Comparing model simulations and experimental observations") that summarizes how our main findings relate to earlier experimental, computational, and network-theoretic work on *C. elegans*.

At **line 946**, we add that **"Our simulation results are broadly consistent with previous experimental and computational studies of the *C. elegans* nervous system. The dominant influence of gap junctions on functional community organization and global synchronization agrees with earlier reports linking electrical coupling to neuronal coherence, chimera-like dynamics, and coordinated locomotion [29,30,40,77]. Likewise, the relative segregation and stronger synchronous responses of locomotion circuits are consistent with evidence that locomotion is governed by a small set of neuronal classes exhibiting coordinated, low-dimensional activity [4,12,40,86]. In contrast, the broader distribution of sensory circuits across functional communities and their more diverse synchronization responses are consistent with the heterogeneous and stimulus-dependent nature of chemosensory and mechanosensory processing [42,53,79]."**

Comment 7: The code used to generate the results should be made publicly available according to the policies of PLOS Computational Biology.

Response: The code used to generate the results has already been made publicly available through the GitHub repository provided in the Data Availability statement of the manuscript. We have rechecked the repository link and confirm that it opens correctly and provides access to the relevant code and data needed to reproduce the analyses.

Response to Reviewer 2

Comment: In their manuscript "Synchronization properties in C. elegans: Relating behavioral circuits to structural and functional neuronal connectivity," the authors construct a computational model of the nematode C. elegans nervous system. In this model, each neuron is represented as a single electrical compartment by a set of differential equations that compute its membrane potential in response to synaptic and gap-junction inputs, based on a published connectivity dataset. The authors examine the response of the nervous system model to prolonged depolarizations in single neurons and focus on the synchronization of neuronal subpopulations. The most interesting result is that neuronal types are not explicitly encoded in the equations, and their categorization emerges from the connectome. The authors also offer three structure-function hypotheses in their conclusions.

This study is interesting, the approach is clear, and the choices are usually explained. My main concerns are that the relevance to behavior is sometimes overstated, and details about the behaviors are not addressed and lost; it is unclear which connectivity dataset(s) are used, and some neurons are ignored, although their function is known.

Response: We thank the reviewer for careful evaluation of our manuscript and for the constructive comments. Below we provide a point-by-point response to each of the comments raised by the reviewer.

Major comments

Comment 1: The term behavior is used loosely in this manuscript. For example, while locomotion and klinotaxis are behaviors, chemosensation and mechanosensation are not. A behavior requires muscle contraction and movement. Focusing on klino-, chemo-, and mechanotaxis, the circuits of these behaviors will include the locomotion circuit, and while mechanotaxis is usually negative (aversive), klino- and chemotaxis in C. elegans can be positive (attractive) or negative (aversive), introducing mixed effects on the activity of the locomotion circuit (note also that forward locomotion could be towards or away from a stimulus). I do not think this complexity can be introduced to the current study, but it should be represented in the introduction and discussion.

Response: We thank the reviewer for this important clarification. We agree that the term behavior was used too broadly in the original manuscript, particularly for chemosensation and mechanosensation, which are sensory modalities rather than complete behaviors by themselves. We have revised the Introduction and Discussion to clarify that these circuits should be interpreted as sensory-processing circuits that can feed into locomotor pathways during taxis behaviors such as chemotaxis, mechanotaxis, and klinotaxis.

In the Introduction of our revised manuscript, at **87** we add that **"Strictly speaking, locomotion and taxis are behaviors that involve motor output requiring muscle contraction and movement, whereas chemosensation and mechanosensation refer to sensory modalities that can drive downstream behavioral responses such as chemotaxis or mechanotaxis [25,26,42,53]. Thus, throughout this study, when we refer to chemosensation and mechanosensation circuits, we mean sensory-processing circuits that can feed into locomotor pathways rather than complete behavior-generating circuits by themselves."**

In the Discussion section at **line 844** we add: **" The distinction between sensory process-**

ing and behavioral motor output is also very important here while interpreting the relation between sensory circuits and locomotion. Taxis behaviors such as chemotaxis, klinotaxis, and mechanotaxis necessarily involve sensory processing (chemosensation, mechanosensation etc.) coupled to locomotor output, and the resulting motor response can be attractive or aversive depending on the stimulus and context [42,43,49,81]. Therefore, our analysis should be interpreted as comparing the synchronization properties of sensory-processing and locomotion-related circuit components, rather than as a complete model of the full sensorimotor transformations underlying each behavior. "

Comment 2: The concepts of synchrony and phase relationships are central to the study. However, calculating those without specific neuronal identities misses the point that coordinated anti-phase activity of antagonistic motor pools is a hallmark of locomotion (and any other rhythmic) behavior. Even a Kuramoto order parameter (Z) that indicates half the neurons are in antiphase with the other half might not represent locomotion behavior if these neurons are not dorsoventral pairs. If such a measure is beyond the scope of the study, the claim of behavior-like output should be weakened and qualified.

Response: We thank the reviewer for this insightful comment. We understand the reviewer's point to concern the rhythmic undulatory nature of *C. elegans* locomotion and its relation to the coordinated, anti-phase activity of dorsoventral motor neuron pairs, and whether our analysis can provide insight into this aspect. Although our original analysis did not explicitly examine synchronization between dorsoventral motor neuron pairs, we have now performed additional analyses in response to the reviewer's comment.

Specifically, we examined synchronization among six locomotion-related motor neuron classes: DA, VA, DB, VB, DD, and VD. The results, presented in Fig. 2, show that the excitatory dorsoventral motor neuron pairs, namely the A-class neurons involved in backward locomotion and the B-class neurons involved in forward locomotion, exhibit a high degree of synchronized activity within their respective groups. In contrast, the D-class inhibitory motor neurons show comparatively weaker synchronization with the A- and B-class neurons, as well as weaker synchronization among themselves.

Although dorsal and ventral motor neurons innervate antagonistic muscles and are often hypothesized to exhibit alternating, anti-phase activity during locomotion, calcium imaging of B-class dorsoventral motor neuron pairs has reported both correlated [5] and anti-correlated [6] activity patterns. More generally, physiological imaging studies of motor neurons have led to apparent discrepancies, with different forms of correlated activity being reported [7]. A recent study by the reviewer [8] further showed that VD and DD inhibitory neurons display alternating activation patterns during high-frequency locomotion, although how these inhibitory dynamics relate to the activity of excitatory motor neurons remains less clear. This makes a direct comparison between our model-based findings and experimental ground truth challenging. Nevertheless, we observe distinct levels of synchronization among the excitatory A- and B-class motor neurons and the inhibitory D-class motor neurons. This is broadly consistent with the known organization of the *C. elegans* locomotor circuit, where A- and B-class motor neurons are associated with backward and forward locomotion, respectively, while D-class GABAergic neurons contribute inhibitory regulation of dorsoventral coordination [7, 9, 8].

To enrich our manuscript with this discussion, at **line 918** we add a subsection **Synchronization of different motor neuron classes in the locomotion circuits** : **"Because rhythmic undulatory locomotion involves coordinated activity across distinct motor neuron classes [38], we performed an additional class-level synchronization analysis on the mo-**

tor neurons in our locomotion circuits including DA, VA, DB, and VB, along with the GABAergic DD, and VD neurons which were reported to be crucial in sustaining rapid dorsoventral alternation [67]; the results are provided in Supplementary Fig. J. This analysis shows that excitatory A- and B-class motor neurons exhibit relatively strong synchronization within their corresponding locomotor groups, whereas inhibitory D-class motor neurons show weaker synchronization with A- and B-class neurons and also among themselves. This result is broadly consistent with the known organization of the locomotor circuit, where A- and B-class motor neurons are primarily associated with backward and forward locomotion, respectively, while D-class GABAergic neurons contribute to inhibitory regulation of dorsoventral coordination [38,40,67]. At the same time, these findings should be interpreted cautiously, since experimental imaging studies have not yielded a single consistent picture of dorsoventral motor neuron dynamics: although dorsal and ventral motor neurons innervate antagonistic muscles and are often hypothesized to exhibit alternating, anti-phase activity during locomotion, calcium imaging of B-class dorsoventral motor neuron pairs has reported correlated activity [86], while other studies have reported different forms of correlated or alternating activity across motor-neuron classes along with several different hypotheses for locomotion [38,67,87]. Thus, our model-based synchronization patterns should not be interpreted as a direct reconstruction of the experimentally observed dorsoventral locomotor rhythm, but rather as a circuit-level indication of how different locomotor motor neuron classes functionally participate in locomotion."

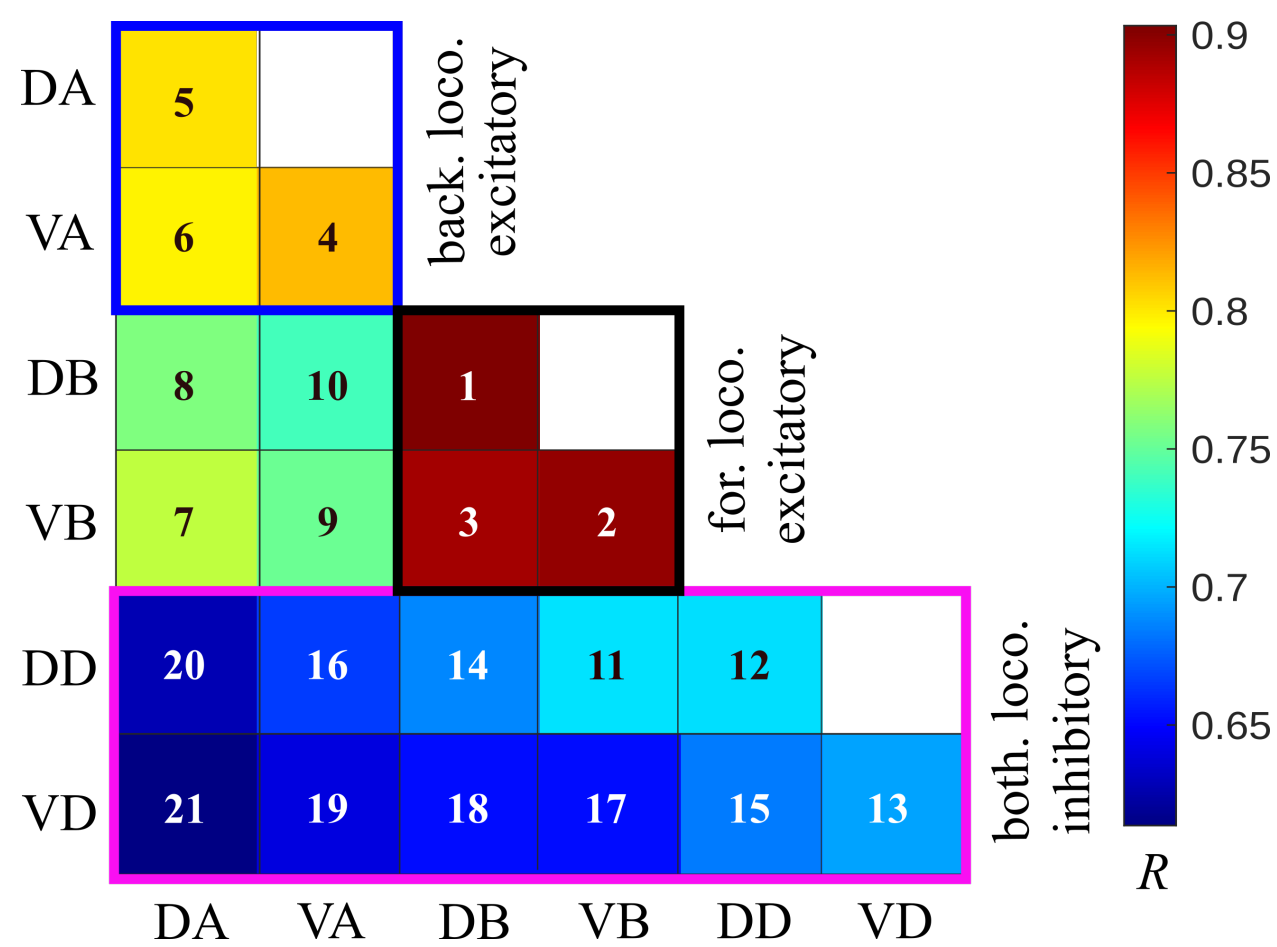

Figure 2: Synchronization between the neurons from six motor neuron classes involved in locomotion. Numbers indicate rank numbers of the entries in descending order.

Comment 3: Clarify which connectivity dataset was used. Some datasets have known limitations (for example, Refs 1-2 are missing the posterior portion of the ventral cord and are a composite of several animals; Cook *et al.* (Ref 52) include an intuitive "interpolation" of that gap). Some information is provided in the "Data Availability" section, citing references 1-6 (only 1-2 provide a reconstructed connectivity dataset). Yet a clear description of the dataset used should be included in the methods section.

Response: We thank the reviewer for this helpful comment. We have now clarified in the Methods section that our analysis uses the reconstructed adult hermaphrodite *C. elegans* connectivity dataset of Varshney et al. [10]. We also explicitly state that this dataset builds on the original White *et*

al. wiring diagram and later updates/reconstructions. We did not use the Cook et al. dataset because some connections in incompletely imaged regions were introduced through anatomy-based extrapolation rather than direct observation.

At **line 119** we add, **" In this study, we use the reconstructed adult hermaphrodite *C. elegans* connectivity dataset of Varshney et al. [2]. This dataset builds on the original wiring diagram of White et al. [1] and incorporates additional reconstructions and subsequent updates, as described in Ref. [2]."**

Comment 4: Some neurons with known function are missing. Notably, the motor neurons of classes AS, DD, VD, and VC could be added to both forward and backward "locomotion circuits". Specifically, the DDs and VDs are among the most connected neurons, as evident in Fig. 1, and are 19 of the 26 GABAergic neurons mentioned in L115.

Response: We thank the reviewer for this enlightening comment. In our study, we considered 'minimal' circuits for forward and backward locomotion by including neurons that are essential for each of these behaviors, while also minimizing the overlap between the two circuits. The behavioral circuits were constructed based on previously published studies, and the locomotion circuits in particular were based on Avila et al., PLOS ONE 2024 [11], in which the AS, DD, VD, and VC neuron classes were excluded on the grounds that they are not strictly required for locomotion [8, 12, 7].

Following the reviewer's suggestion, we repeated our analysis using updated forward and backward locomotion circuits that included all four of these neuronal classes. The neurons in the modified circuits are listed in Table 3, where the added neurons, shown in *red*, were taken from [7]. The synchronization patterns remain largely similar after this update, as shown by comparing the left and right columns of Fig. 3. Moreover, when we compare the distribution of neurons in the updated locomotion circuits across the FCs, we still observe segregation into a small number of FCs, although this segregation is somewhat diluted due to the inclusion of additional neurons; see Table 4. This dilution can be attributed to the substantial overlap introduced by adding the four neuronal classes to both the forward and backward locomotion circuits. Specifically, the updated circuits share 35 neurons, comprising 11 AS, 5 VC, 6 DD, and 13 VD neurons. This overlap is larger than the total number of distinct neurons in either of the original circuits considered in our study, which contained 24 neurons in the forward locomotion circuit and 31 neurons in the backward locomotion circuit.

In summary, including these four neuronal classes, most of which have limited or incompletely understood roles in locomotion, increases the overlap between the forward and backward locomotion circuits while leaving the main findings of our study largely unaffected. We therefore retained the original locomotion circuit definitions in the main analysis and included the results obtained using the extended circuits in the Supplementary Material.

We add make the following changes in our revised manuscript to address this issue. At **line 168**, after describing the locomotion circuits we mention that – **"Following previous modeling studies of the locomotion network [36,38,67,68], the AS, DD, VD, and VC neuronal classes are not included in our main definitions of the forward and backward locomotion circuits. This choice allows us to focus on minimal behavior-specific locomotion circuits with limited overlap between them; nevertheless, we also repeated the analysis using extended locomotion circuits that include these neuronal classes [38]. The expanded forward and backward locomotion circuits exhibit substantially greater overlap between them, while the principal synchronization patterns and the main conclusions of our study remain largely unchanged (see Table E, Fig. L and Table F in the Supplementary Material)."**

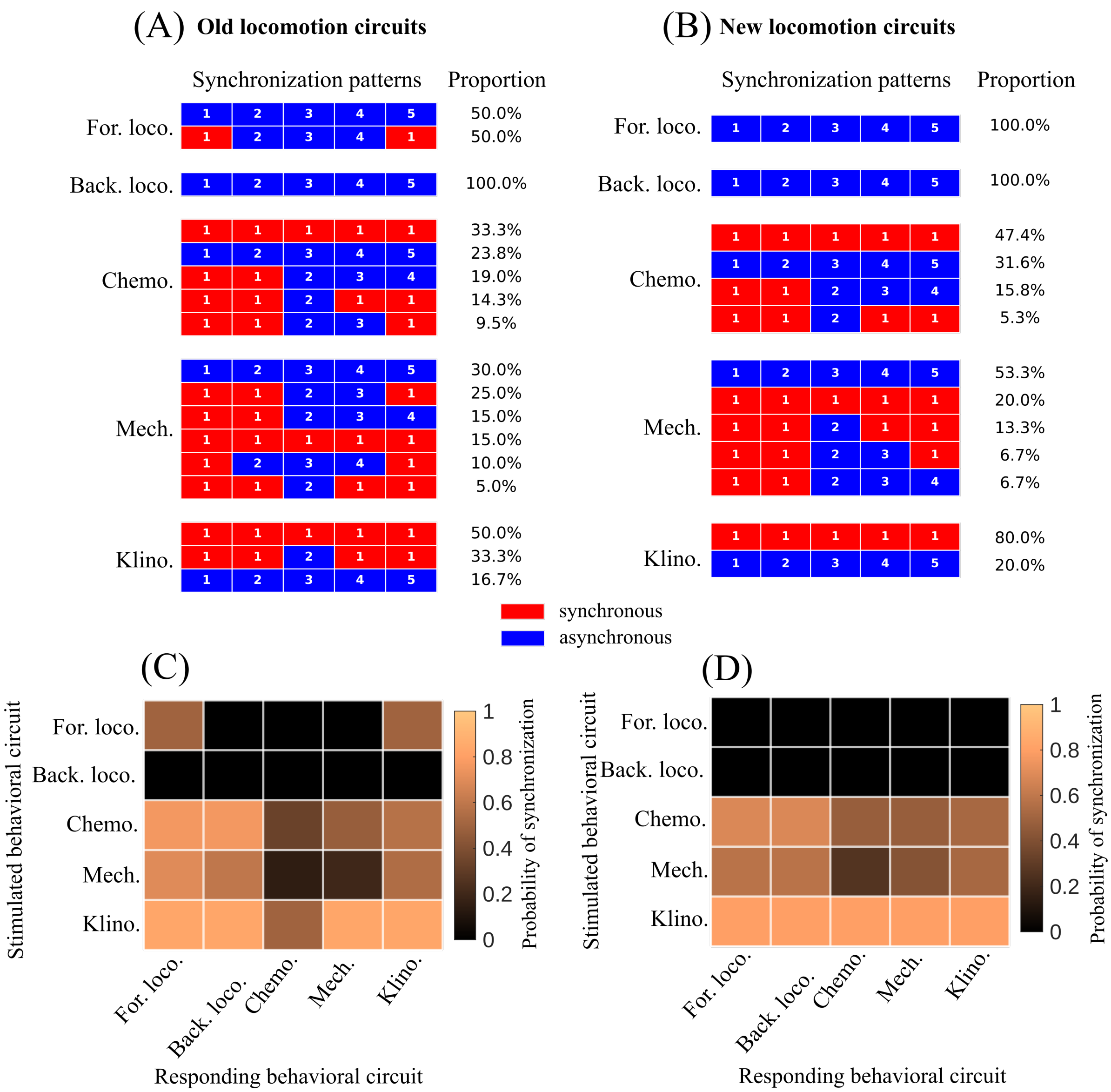

Figure 3: Comparison between the synchronization patterns with old and new locomotion circuits.

Table 3: The extended locomotion circuits

Circuit	Neurons
Forward locomotion (61)	AS1, AS2, AS3, AS4, AS5, AS6, AS7, AS8, AS9, AS10, AS11, AVBL, AVBR, DB1, DB2, DB3, DB4, DB5, DB6, DB7, DD1, DD2, DD3, DD4, DD5, DD6, DVA, PVCL, PVCR, RIBL, RIBR, RID, VB1, VB2, VB3, VB4, VB5, VB6, VB7, VB8, VB9, VB10, VB11, VC1, VC2, VC3, VC4, VC5, VD1, VD2, VD3, VD4, VD5, VD6, VD7, VD8, VD9, VD10, VD11, VD12, VD13
Backward locomotion (68)	AIBL, AIBR, AS1, AS2, AS3, AS4, AS5, AS6, AS7, AS8, AS9, AS10, AS11, AVAL, AVAR, AVDL, AVDR, AVEL, AVER, DA1, DA2, DA3, DA4, DA5, DA6, DA7, DA8, DA9, DD1, DD2, DD3, DD4, DD5, DD6, DVA, RID, RIML, RIMR, VA1, VA2, VA3, VA4, VA5, VA6, VA7, VA8, VA9, VA10, VA11, VA12, VC1, VC2, VC3, VC4, VC5, VD1, VD2, VD3, VD4, VD5, VD6, VD7, VD8, VD9, VD10, VD11, VD12, VD13

Table 4: Functional community-wise distribution of old and new locomotion circuits across Type I and Type II functional community partitions

Type I	Forward loco.		Backward loco.		Type II	Forward loco.		Backward loco.	
	Old (24)	New (61)	Old (31)	New (68)		Old (24)	New (61)	Old (31)	New (68)
**FC 1 (75)**	22 (92%)	35 (57%)	23 (74%)	36 (53%)	**FC 1 (53)**	4	14	23 (74%)	33 (49%)
**FC 2 (67)**	2	15	3	16	**FC 2 (84)**	20 (83%)	36 (59%)	3	19
**FC 3 (52)**	0	6	5	11	**FC 3 (57)**	0	6	5	11
**FC 4 (45)**	0	2	0	2	**FC 4 (45)**	0	2	0	2
**FC 5 (27)**	0	2	0	2	**FC 5 (27)**	0	2	0	2
**FC 6 (6)**	0	1	0	1	**FC 6 (6)**	0	1	0	1

The table shows how neurons from the old and new versions of the forward and backward locomotion circuits are distributed across the identified functional communities under Type I and Type II functional community partitions.

Minor comments

Comment: (Most of those comments are examples of similar issues throughout the text and should be applied everywhere.)
Results should be written in the past tense.
Equations are clearly explained.
The discussion is well developed and well written.

Response: Thanks for the feedback.

Comment 1: L9-10: Add citations of N. Cohen and S. Flavel for computational models and whole-brain recording, respectively.

Response: The references (Refs. [10-11] for computational models, and Refs. [15-16] for whole-brain recording) have been added.

Comment 2: L46: ref 25 seems to be in the wrong place, or missing citation for computational approach earlier in the sentence.

Response: Placing of this reference has been fixed.

Comment 3: L49-50: Cited references demonstrate the existence of action potentials in body wall muscle and some neurons, contrary to the text. Correct the text to be more accurate.

Response: We fixed this. We reframed our sentence as: "However, this modeling choice is biologically questionable, as neural signaling in *C. elegans* is predominantly mediated by graded membrane-potential changes [31], although action potentials have lately been identified in a small number of specialized neurons and in body-wall muscles [32,33]".

Comment 4: L122: Motor neurons in C. elegans generate the coordinated activity; it is relayed to the muscle by the muscle arms (see Haspel et al., 2020; Elegantly)

Response: We have modified the text as: "motor neurons generate coordinated activity patterns that are relayed to the body-wall muscles through neuromuscular contacts formed by muscle arms, thereby producing behavioral output."

Comment 5: L696: behavior is not a "property". Edit to "…behavior emerges from structure and function."

Response: Thanks. Fixed.

Comment 6: L751: Edit. There is no one-to-one correspondence.

Response: We have edited it to: "We find that the forward and backward locomotion circuits are largely segregated into two FCs"

Comment 7: L892-894: The hypotheses are insightful. Hypothesis #3 could be less study-specific, for example: "Neurons that are not connected by gap junctions could serve as organizers that elicit synchronized activity in downstream circuits.

Response: We appreciate this suggestion and included it.

Comment 8: Fig. 1, 4: The color scheme is inconsistent between figures. Red (e.g., motor) and magenta (e.g., sensory) colors are too close and hard to distinguish.

Response: These issues have been taken care of by choosing appropriate colors.

Comment 9: Fig. 3B: Should "I ext" be "Re(lambda i)", as in A?

Response: The missing y-label has been fixed.

Comment 10: Fig 10B: missing axis titles.

Response: Fixed. Axis titles added.

References

[1] Fenyves BG, Szilágyi GS, Vassy Z, Sőti C, Csermely P. Synaptic polarity and sign-balance prediction using gene expression data in the Caenorhabditis elegans chemical synapse neuronal connectome network. PLOS Computational Biology. 2020;16(12):e1007974.

[2] Kunert J, Shlizerman E, Kutz JN. Low-dimensional functionality of complex network dynamics: Neurosensory integration in the Caenorhabditis elegans connectome. Physical Review E. 2014;89(5):052805.

[3] Kunert-Graf JM, Shlizerman E, Walker A, Kutz JN. Multistability and long-timescale transients encoded by network structure in a model of C. elegans connectome dynamics. Frontiers in Computational Neuroscience. 2017;11:53.

[4] Kunert JM, Proctor JL, Brunton SL, Kutz JN. Spatiotemporal feedback and network structure drive and encode Caenorhabditis elegans locomotion. PLoS Computational Biology. 2017;13(1):e1005303.

[5] Haspel G, O'Donovan MJ, Hart AC. Motoneurons dedicated to either forward or backward locomotion in the nematode Caenorhabditis elegans. Journal of Neuroscience. 2010;30(33):11151-6.

[6] Wen Q, Po MD, Hulme E, Chen S, Liu X, Kwok SW, et al. Proprioceptive coupling within motor neurons drives C. elegans forward locomotion. neuron. 2012;76(4):750-61.

[7] Gjorgjieva J, Biron D, Haspel G. Neurobiology of Caenorhabditis elegans locomotion: where do we stand? Bioscience. 2014;64(6):476-86.

[8] Deng L, Denham JE, Arya C, Yuval O, Cohen N, Haspel G. Inhibition underlies fast undulatory locomotion in Caenorhabditis elegans. Eneuro. 2021;8(2).

[9] Zhen M, Samuel AD. C. elegans locomotion: small circuits, complex functions. Current Opinion in Neurobiology. 2015;33:117-26.

[10] Varshney LR, Chen BL, Paniagua E, Hall DH, Chklovskii DB. Structural properties of the Caenorhabditis elegans neuronal network. PLoS Computational Biology. 2011;7(2):e1001066.

[11] Avila B, Serafino M, Augusto P, Zimmer M, Makse HA. Fibration symmetries and cluster synchronization in the Caenorhabditis elegans connectome. Plos One. 2024;19(4):e0297669.

[12] Haspel G, Severi KE, Fauci LJ, Cohen N, Tytell ED, Morgan JR. Resilience of neural networks for locomotion. The Journal of Physiology. 2021;599(16):3825-40.
