# Supplementary material for: Synchronization properties in C. elegans: Relating behavioral circuits to structural and functional neuronal connectivity
Source: PLoS Comput Biol. 2026 Sep 15;22(9):e1014152. doi: 10.1371/journal.pcbi.1014152 (PMC13588538; doi:10.1371/journal.pcbi.1014152)
Supplement: Peer review history

# Peer review: Decision Letter 0

**DOI:** 10.1371/journal.pcbi.1014152.r001

**Authors:** Lyle J. Graham, Barbara Webb

20 May 2026

Synchronization properties in C. elegans: Relating behavioral circuits to structural and functional neuronal connectivity

PLOS Computational Biology

Dear Dr. Davidsen,

Thank you for submitting your manuscript to PLOS Computational Biology. After careful consideration, we feel that it has merit but does not fully meet PLOS Computational Biology's publication criteria as it currently stands. Therefore, we invite you to submit a revised version of the manuscript that addresses the points raised during the review process.

Please submit your revised manuscript by Jul 20 2026 11:59PM. If you will need more time than this to complete your revisions, please reply to this message or contact the journal office at ploscompbiol@plos.org. When you're ready to submit your revision, log on to https://www.editorialmanager.com/pcompbiol/ and select the 'Submissions Needing Revision' folder to locate your manuscript file.

Please include the following items when submitting your revised manuscript:

* A letter that responds to each point raised by the editor and reviewer(s). You should upload this letter as a separate file labeled 'Response to Reviewers'. This file does not need to include responses to formatting updates and technical items listed in the 'Journal Requirements' section below.

* A marked-up copy of your manuscript that highlights changes made to the original version. You should upload this as a separate file labeled 'Revised Manuscript with Track Changes'.

* An unmarked version of your revised paper without tracked changes. You should upload this as a separate file labeled 'Manuscript'.

We look forward to receiving your revised manuscript.

Kind regards,

Barbara Webb

Academic Editor

PLOS Computational Biology

Lyle Graham

Section Editor

PLOS Computational Biology

**Journal Requirements:**

1) We ask that a manuscript source file is provided at Revision. Please upload your manuscript file as a .doc, .docx, .rtf or .tex. If you are providing a .tex file, please upload it under the item type u2018LaTeX Source Fileu2019 and leave your .pdf version as the item type u2018Manuscriptu2019.

2) Please upload all main figures as separate Figure files in .tif or .eps format. For more information about how to convert and format your figure files please see our guidelines:

https://journals.plos.org/ploscompbiol/s/figures

3) We notice that your supplementary Figures, and Tables are included in the manuscript file. Please remove them and upload them with the file type 'Supporting Information'. Please ensure that each Supporting Information file has a legend listed in the manuscript after the references list.

Note: If the reviewer comments include a recommendation to cite specific previously published works, please review and evaluate these publications to determine whether they are relevant and should be cited. There is no requirement to cite these works unless the editor has indicated otherwise.

**Reviewers' comments:**

**Comments to the Authors:**

**Please note that one review is uploaded as an attachment.**

Reviewer #1: The review is uploaded as an attachment.

Reviewer #2: In their manuscript “Synchronization properties in C. elegans: Relating behavioral circuits to structural and functional neuronal connectivity,” the authors construct a computational model of the nematode C. elegans nervous system. In this model, each neuron is represented as a single electrical compartment by a set of differential equations that compute its membrane potential in response to synaptic and gap-junction inputs, based on a published connectivity dataset. The authors examine the response of the nervous system model to prolonged depolarizations in single neurons and focus on the synchronization of neuronal subpopulations. The most interesting result is that neuronal types are not explicitly encoded in the equations, and their categorization emerges from the connectome. The authors also offer three structure-function hypotheses in their conclusions.

This study is interesting, the approach is clear, and the choices are usually explained. My main concerns are that the relevance to behavior is sometimes overstated, and details about the behaviors are not addressed and lost; it is unclear which connectivity dataset(s) are used, and some neurons are ignored, although their function is known.

Major comments:

The term behavior is used loosely in this manuscript. For example, while locomotion and klinotaxis are behaviors, chemosensation and mechanosensation are not. A behavior requires muscle contraction and movement. Focusing on klino-, chemo-, and mechanotaxis, the circuits of these behaviors will include the locomotion circuit, and while mechanotaxis is usually negative (aversive), klino- and chemotaxis in C. elegans can be positive (attractive) or negative (aversive), introducing mixed effects on the activity of the locomotion circuit (note also that forward locomotion could be towards or away from a stimulus). I do not think this complexity can be introduced to the current study, but it should be represented in the introduction and discussion.

The concepts of synchrony and phase relationships are central to the study. However, calculating those without specific neuronal identities misses the point that coordinate anti-phase activity of antagonistic motor pools is a hallmark of locomotion (and any other rhythmic) behavior. Even a Kuramoto order parameter (Z) that indicates half the neurons are in antiphase with the other half might not represent locomotion behavior if these neurons are not dorsoventral pairs. If such a measure is beyond the scope of the study, the claim of behavior-like output should be weakened and qualified.

Clarify which connectivity dataset was used. Some datasets have known limitations (for example, Refs 1-2 are missing the posterior portion of the ventral cord and are a composite of several animals; Cook et al. (Ref 52) include an intuitive “interpolation” of that gap). Some information is provided in the “Data Availability” section, citing references 1-6 (only 1-2 provide a reconstructed connectivity dataset). Yet a clear description of the dataset used should be included in the methods section.

Some neurons with known function are missing. Notably, the motor neurons of classes AS, DD, VD, and VC could be added to both forward and backward “locomotion circuits”. Specifically, the DDs and VDs are among the most connected neurons, as evident in Fig. 1, and are 19 of the 26 GABAergic neurons mentioned in L115.

Minor comments:

(Most of those comments are examples of similar issues throughout the text and should be applied everywhere.)

Results should be written in the past tense.

Equations are clearly explained.

The discussion is well developed and well written.

By lines:

L9-10: Add citations of N. Cohen and S. Flavel for computational models and whole-brain recording, respectively.

L46: ref 25 seems to be in the wrong place, or missing citation for computational approach earlier in the sentence.

L49-50: Cited references demonstrate the existence of action potentials in body wall muscle and some neurons, contrary to the text. Correct the text to be more accurate.

L122: Motor neurons in C. elegans generate the coordinated activity; it is relayed to the muscle by the muscle arms (see Haspel et al., 2020; Elegantly)

L696: behavior is not a “property”. Edit to “…behavior emerges from structure and function.”

L751: Edit. There is no one-to-one correspondence.

L892-894: The hypotheses are insightful. Hypothesis #3 could be less study-specific, for example: “Neurons that are not connected by gap junctions could serve as organizers that elicit synchronized activity in downstream circuits.

Figures

Fig. 1, 4: The color scheme is inconsistent between figures. Red (e.g., motor) and magenta (e.g., sensory) colors are too close and hard to distinguish.

Fig. 3B: Should “I ext” be “Re(lambda i)”, as in A?

Fig 10B: missing axis titles.

**********

**Have the authors made all data and (if applicable) computational code underlying the findings in their manuscript fully available?**

The PLOS Data policy requires authors to make all data and code underlying the findings described in their manuscript fully available without restriction, with rare exception (please refer to the Data Availability Statement in the manuscript PDF file). The data and code should be provided as part of the manuscript or its supporting information, or deposited to a public repository. For example, in addition to summary statistics, the data points behind means, medians and variance measures should be available. If there are restrictions on publicly sharing data or code —e.g. participant privacy or use of data from a third party—those must be specified.

Reviewer #1: **No:**  The authors included a link to a GitHub repo but the link does not work.

Reviewer #2: Yes

**********

PLOS authors have the option to publish the peer review history of their article (what does this mean?). If published, this will include your full peer review and any attached files.

If you choose “no”, your identity will remain anonymous but your review may still be made public.

**Do you want your identity to be public for this peer review?**  For information about this choice, including consent withdrawal, please see our Privacy Policy.

Reviewer #1: **Yes:**  Megan Morrison

Reviewer #2: **Yes:**  Gal Haspel

[NOTE: If reviewer comments were submitted as an attachment file, they will be attached to this email and accessible via the submission site. Please log into your account, locate the manuscript record, and check for the action link "View Attachments". If this link does not appear, there are no attachment files.]

**Figure resubmission:**

While revising your submission, we strongly recommend that you use PLOS’s NAAS tool (https://ngplosjournals.pagemajik.ai/artanalysis) to test your figure files. NAAS can convert your figure files to the TIFF file type and meet basic requirements (such as print size, resolution), or provide you with a report on issues that do not meet our requirements and that NAAS cannot fix.

After uploading your figures to PLOS’s NAAS tool - https://ngplosjournals.pagemajik.ai/artanalysis, NAAS will process the files provided and display the results in the "Uploaded Files" section of the page as the processing is complete. If the uploaded figures meet our requirements (or NAAS is able to fix the files to meet our requirements), the figure will be marked as "fixed" above. If NAAS is unable to fix the files, a red "failed" label will appear above. When NAAS has confirmed that the figure files meet our requirements, please download the file via the download option, and include these NAAS processed figure files when submitting your revised manuscript.

**Reproducibility:**

To enhance the reproducibility of your results, we recommend that authors of applicable studies deposit laboratory protocols in protocols.io, where a protocol can be assigned its own identifier (DOI) such that it can be cited independently in the future. Additionally, PLOS ONE offers an option to publish peer-reviewed clinical study protocols. Read more information on sharing protocols at https://plos.org/protocols?utm_medium=editorial-email&utm_source=authorletters&utm_campaign=protocols

Attachment Submitted filename: Review_PLOS_Synchronization.pdf

# Author comment: Author response to Decision Letter 1

**DOI:** 10.1371/journal.pcbi.1014152.r002

17 Jul 2026

Attachment Submitted filename: Response to Reviewers.pdf

# Peer review: Decision Letter 1

**DOI:** 10.1371/journal.pcbi.1014152.r003

**Authors:** Lyle J. Graham, Barbara Webb

26 Aug 2026

Dear Prof. Davidsen,

We are pleased to inform you that your manuscript 'Synchronization properties in C. elegans: Relating behavioral circuits to structural and functional neuronal connectivity' has been provisionally accepted for publication in PLOS Computational Biology.

Before your manuscript can be formally accepted you will need to complete some formatting changes, which you will receive in a follow up email. A member of our team will be in touch with a set of requests.

Please note that your manuscript will not be scheduled for publication until you have made the required changes, so a swift response is appreciated.

IMPORTANT: The editorial review process is now complete. PLOS will only permit corrections to spelling, formatting or significant scientific errors from this point onwards. Requests for major changes, or any which affect the scientific understanding of your work, will cause delays to the publication date of your manuscript.

Should you, your institution's press office or the journal office choose to press release your paper, you will automatically be opted out of early publication. We ask that you notify us now if you or your institution is planning to press release the article. All press must be co-ordinated with PLOS.

Thank you again for supporting Open Access publishing; we are looking forward to publishing your work in PLOS Computational Biology.

Best regards,

Barbara Webb

Academic Editor

PLOS Computational Biology

Lyle Graham

Section Editor

PLOS Computational Biology

***********************************************************

Reviewer's Responses to Questions

**Comments to the Authors:**

**Please note here if the review is uploaded as an attachment.**

Reviewer #1: The authors have responded to all of my questions and concerns and have done substantial new work to address the points that I raised.

Reviewer #2: As noted by the authors, the revision has greatly improved the manuscript. The authors added new analyses that include more (about twofold!) motoneuron classes and address the known functions of motoneuron classes. They also clarified parts of the original text and added behavior-minded insights to the introduction and discussion. The new analyses and insights are on point. For example, the explicit insights about the distinct roles of GJ and chemical synapses. The new discussion sections “Comparing model simulations and experimental observations" (following reviewer 1's suggestion) and “Synchronization of different motor neuron classes in the locomotion circuits” provide context to the findings and insights.

**********

**Have the authors made all data and (if applicable) computational code underlying the findings in their manuscript fully available?**

The PLOS Data policy requires authors to make all data and code underlying the findings described in their manuscript fully available without restriction, with rare exception (please refer to the Data Availability Statement in the manuscript PDF file). The data and code should be provided as part of the manuscript or its supporting information, or deposited to a public repository. For example, in addition to summary statistics, the data points behind means, medians and variance measures should be available. If there are restrictions on publicly sharing data or code —e.g. participant privacy or use of data from a third party—those must be specified.

Reviewer #1: Yes

Reviewer #2: Yes

**********

PLOS authors have the option to publish the peer review history of their article (what does this mean?). If published, this will include your full peer review and any attached files.

If you choose “no”, your identity will remain anonymous but your review may still be made public.

**Do you want your identity to be public for this peer review?**  For information about this choice, including consent withdrawal, please see our Privacy Policy.

Reviewer #1: No

Reviewer #2: **Yes:**  Gal Haspel

# Editor report: Acceptance letter

**DOI:** 10.1371/journal.pcbi.1014152.r004

**Authors:** Lyle J. Graham, Barbara Webb

PCOMPBIOL-D-26-00649R1

Synchronization properties in C. elegans: Relating behavioral circuits to structural and functional neuronal connectivity

Dear Dr Davidsen,

I am pleased to inform you that your manuscript has been formally accepted for publication in PLOS Computational Biology. Your manuscript is now with our production department and you will be notified of the publication date in due course.

The corresponding author will soon be receiving a typeset proof for review, to ensure errors have not been introduced during production. Please review the PDF proof of your manuscript carefully, as this is the last chance to correct any errors. Please note that major changes, or those which affect the scientific understanding of the work, will likely cause delays to the publication date of your manuscript.

Soon after your final files are uploaded, unless you have opted out, the early version of your manuscript will be published online. The date of the early version will be your article's publication date. The final article will be published to the same URL, and all versions of the paper will be accessible to readers.

For Research, Software, and Methods articles, you will receive an invoice from PLOS for your publication fee after your manuscript has reached the completed accept phase. If you receive an email requesting payment before acceptance or for any other service, this may be a phishing scheme. Learn how to identify phishing emails and protect your accounts at https://explore.plos.org/phishing.

Thank you again for supporting PLOS Computational Biology and open-access publishing. We are looking forward to publishing your work!

With kind regards,

Sharmila Kamatchi

PLOS Computational Biology | Carlyle House, Carlyle Road, Cambridge CB4 3DN | United Kingdom ploscompbiol@plos.org | Phone +44 (0) 1223-442824 | ploscompbiol.org | @PLOSCompBiol
